# Supplementary material for: Effect of Copper Antifouling Paint on Marine Degradation of Polypropylene: Uneven Distribution of Microdebris between Nagasaki Port and Goto Island, Japan
Source: Molecules. 2024 Mar 6;29(5):1173. doi: 10.3390/molecules29051173 (PMC10934280; doi:10.3390/molecules29051173)
Supplement: Supplementary file 1 [file molecules-29-01173-s001.zip › molecules-2857748-supplementary.pdf]

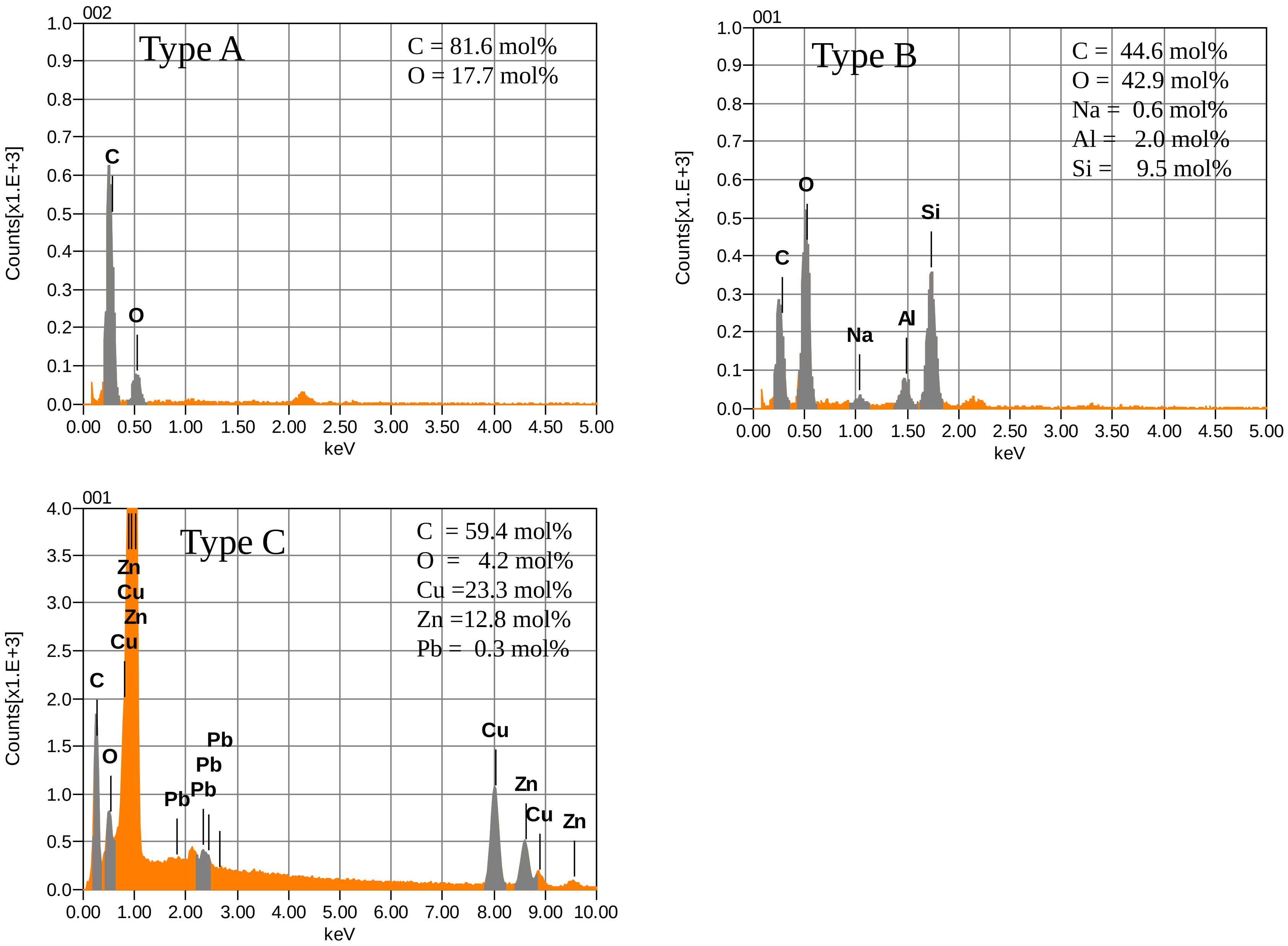

Figure S1 EDX analysis of samples retrieved by the sea: Type A: MP. Type B: Si based paint particle. Type C: Cu base paint particle Notes: Measuring elements except gold coating material.

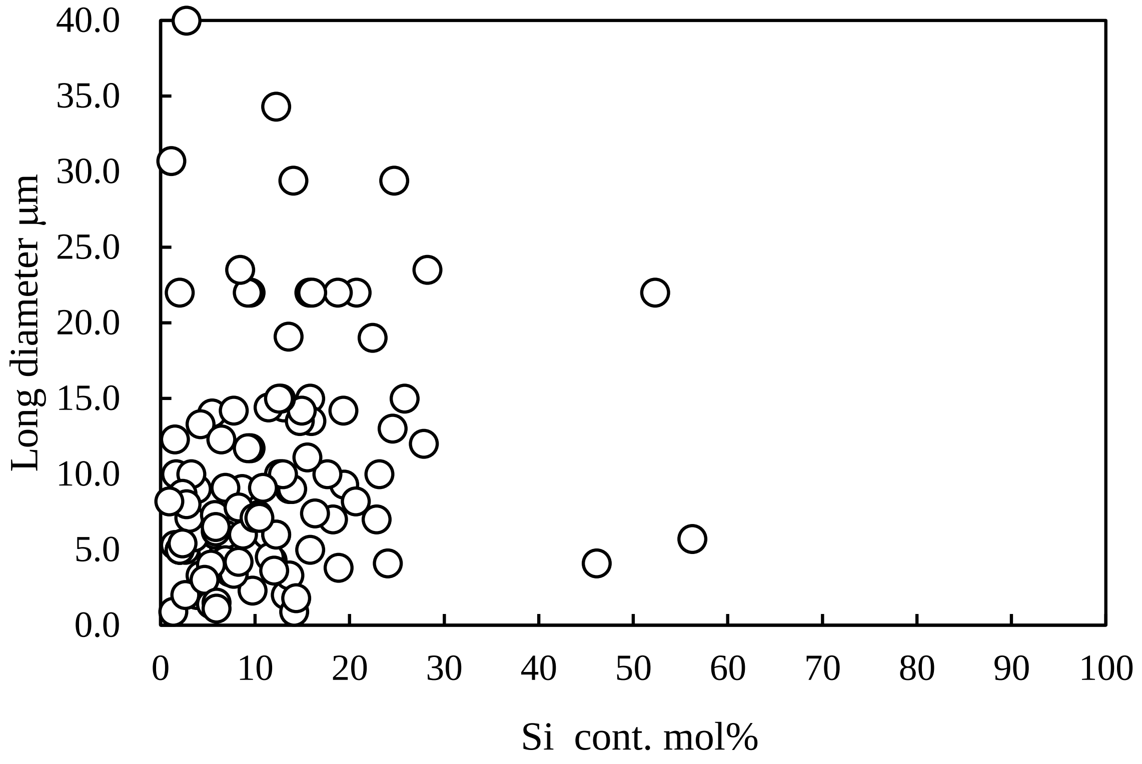

Figure S2 Relationship between Si content ratio and long diameter of type B (Si based paint particle) samples retrieved from the sea.

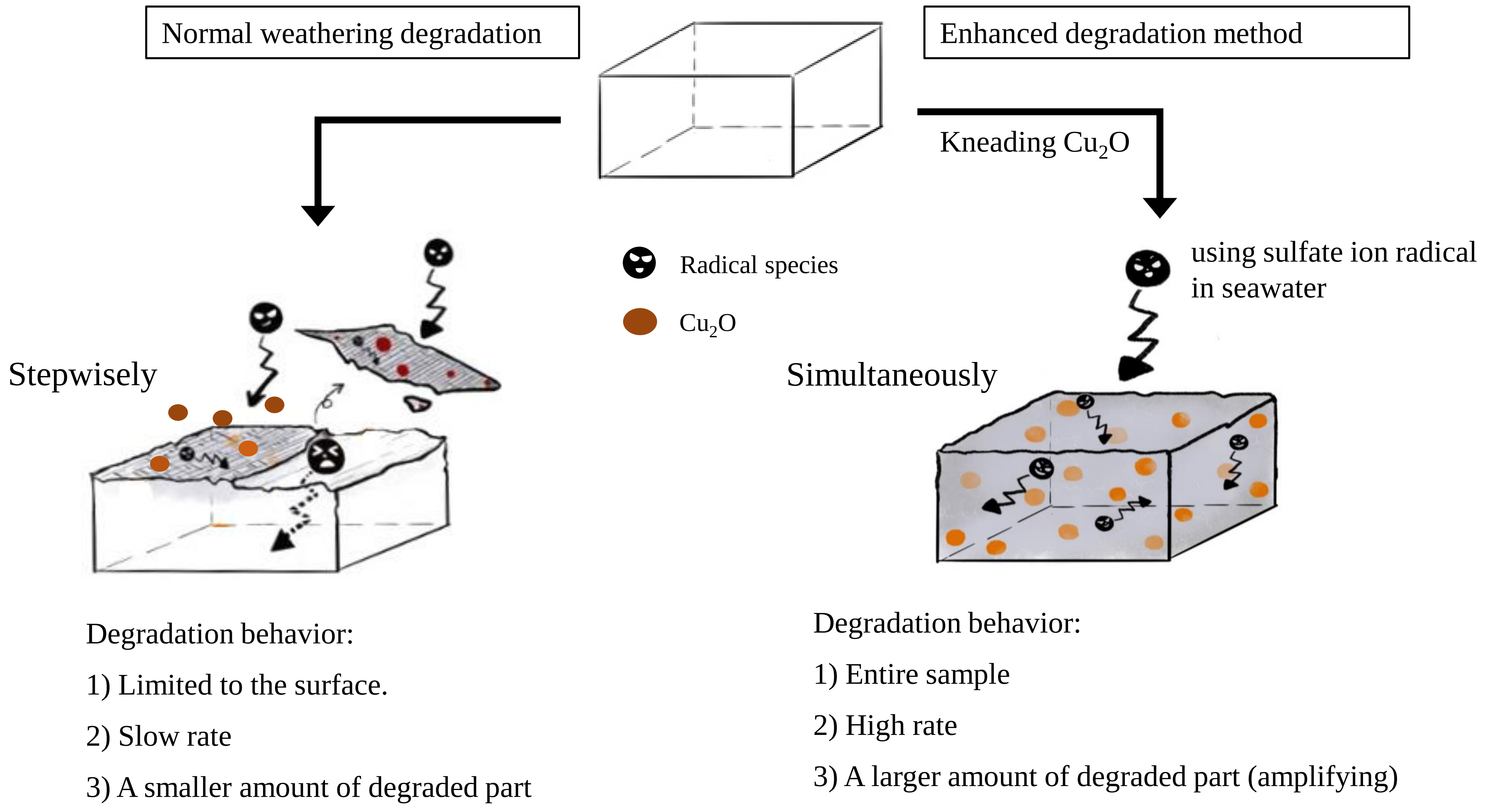

Figure S3 Comparison of degradation behavior between normal weathering degradation and enhanced degradation in seawater.

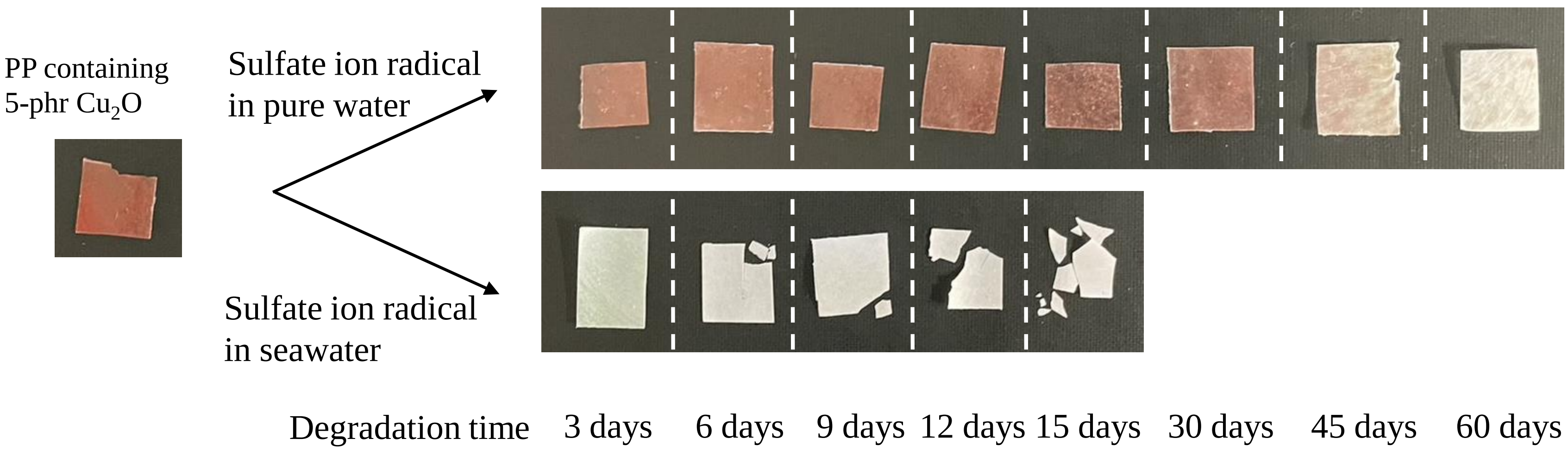

Figure S4 Color change of PP containing 5-phr Cu_2O film by the enhanced degradation method..

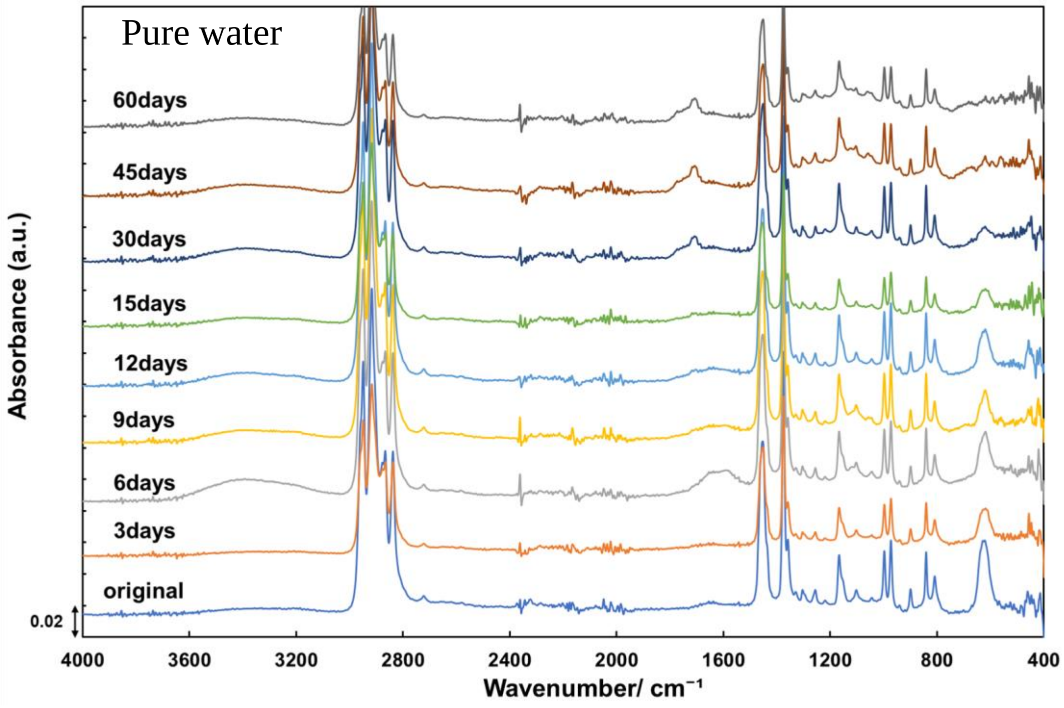

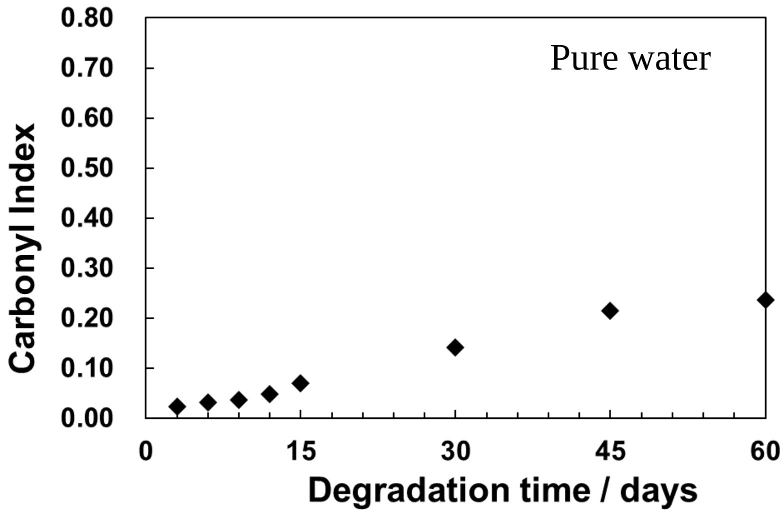

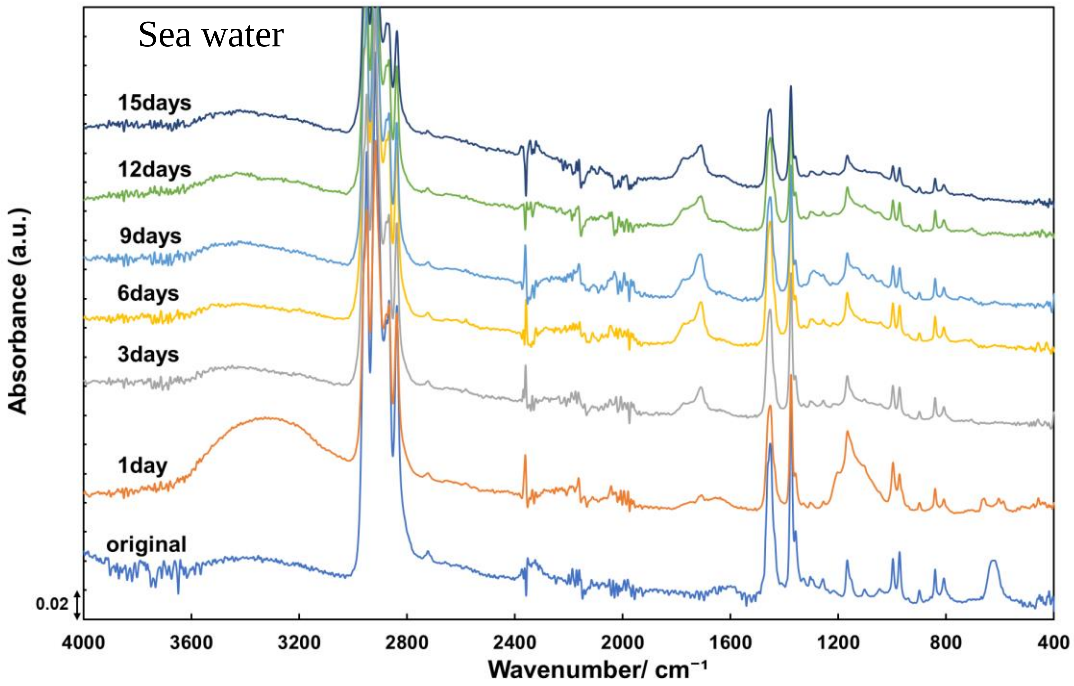

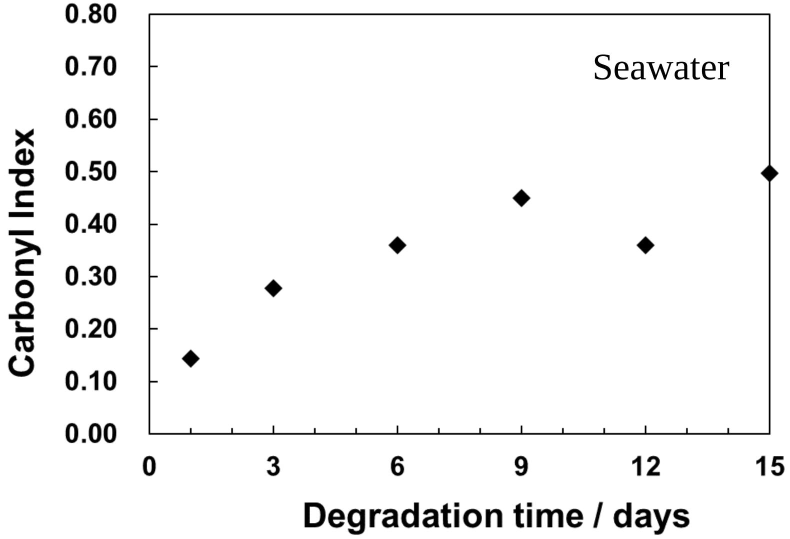

Figure S5 Degradation time dependences of FT-IR spectra and carbonyl index values of degraded PP containing 5-phr Cu_2O by the enhanced degradation method in pure and seawater.

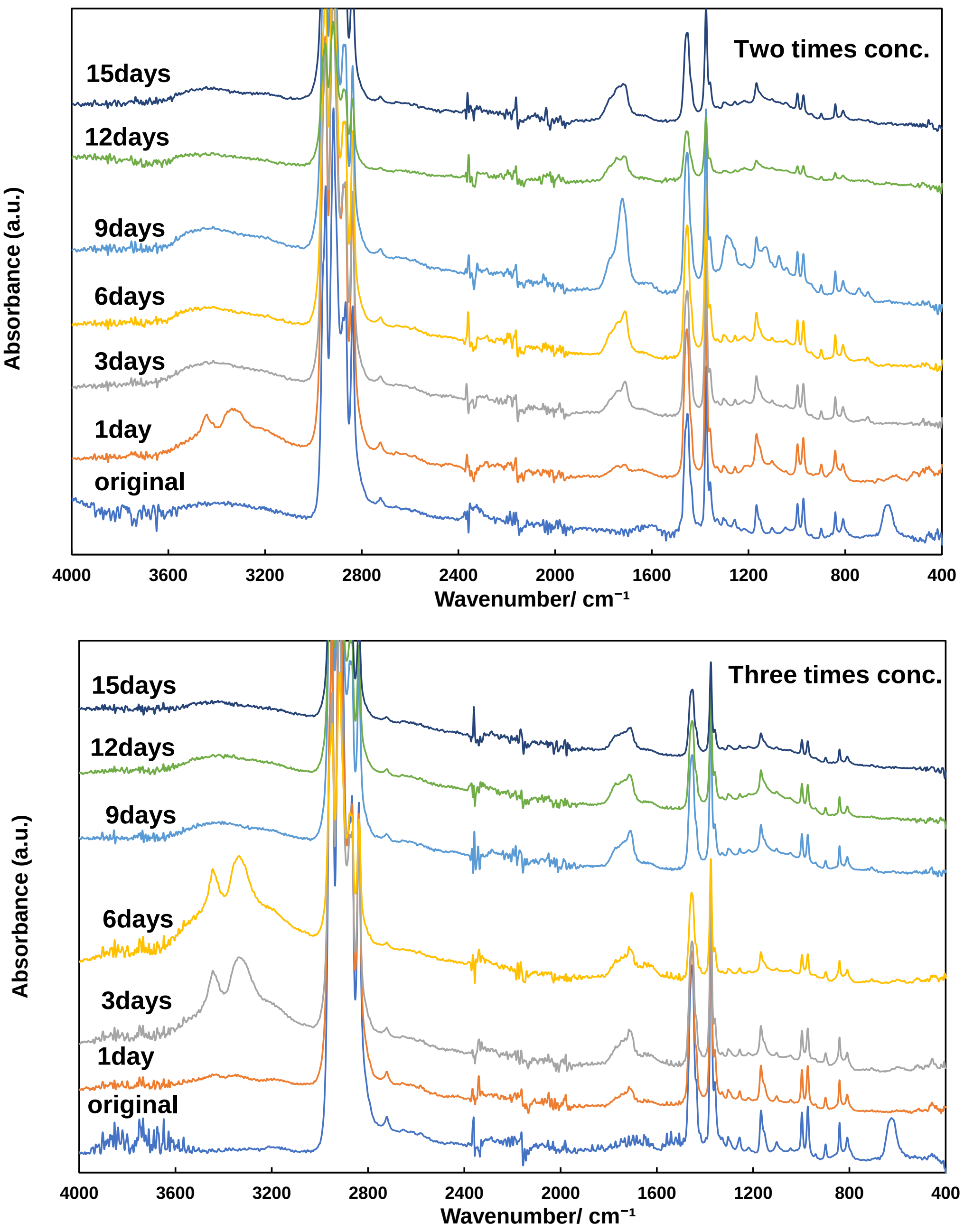

Figure S6 Degradation time dependences of FT-IR spectra of degraded PP containing 5-phr Cu_2O by the enhanced degradation method in two and three times salinity concentrations of seawater.

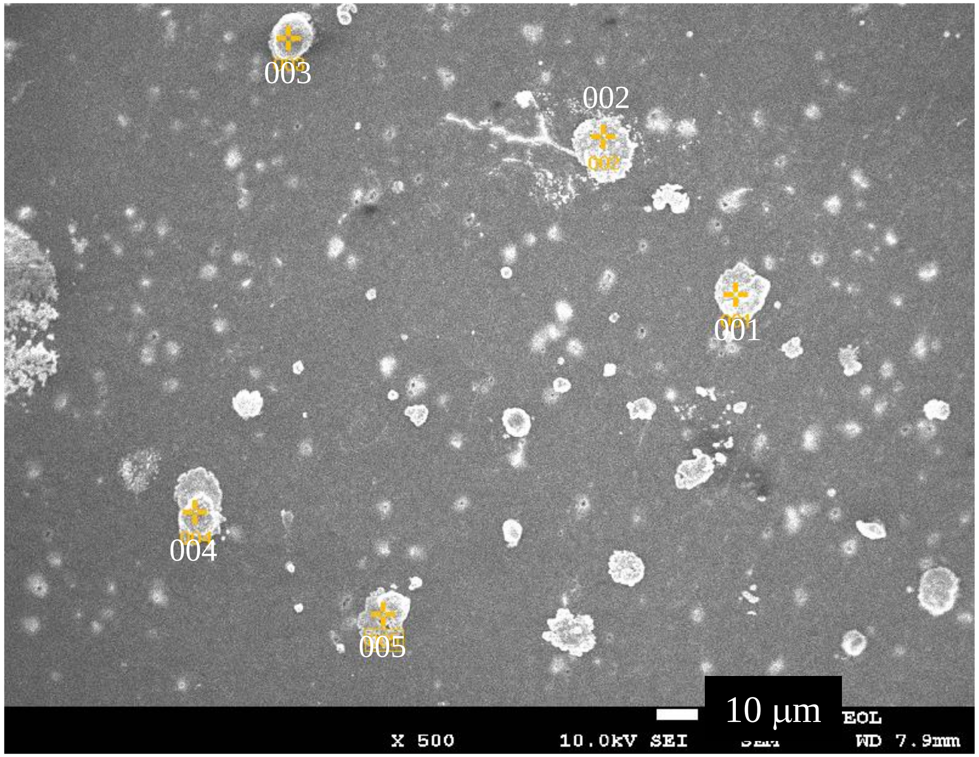

	O (mol%)	C (mol%)	Cl (mol%)	Cu (mol%)	Hg (mol%)
001					
002	34.76		17.15	48.09	
003	35.16		24.58	40.26	
004	36.36		22.25	41.39	
005	9.48	59.43	24.69	3.09	3.30

Figure S7 SEM/EDX analysis of degraded PP containing 5-phr Cu_2O by the enhanced degradation method in seawater for 3 days.

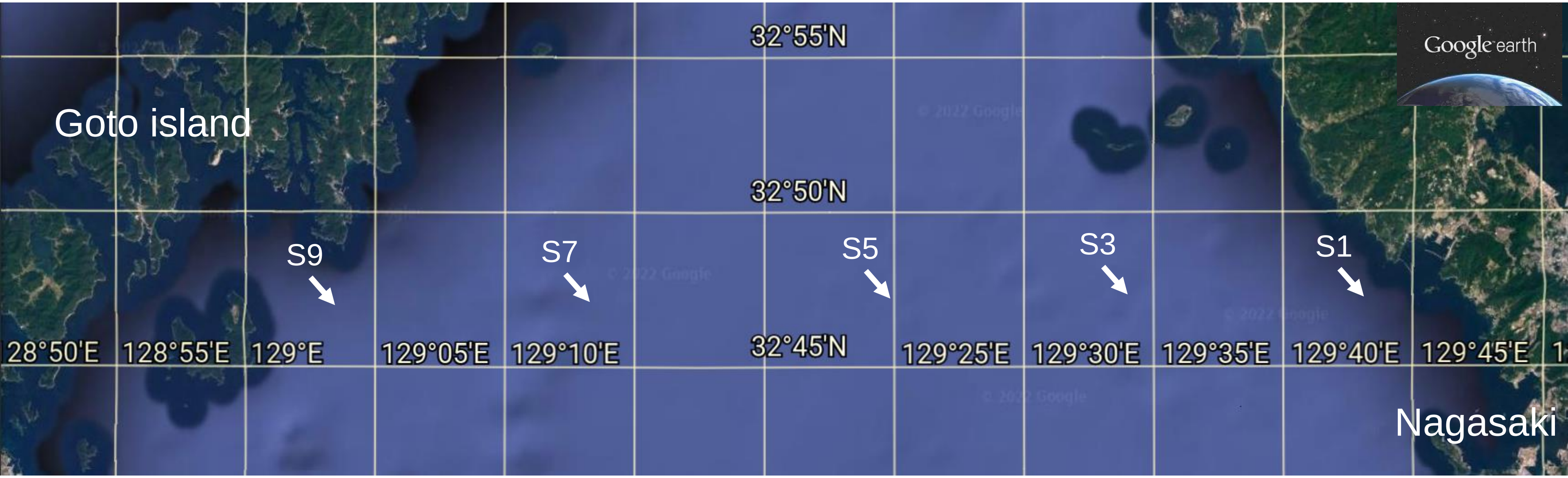

Figure S8 Sampling station number and coordinates.
https://earth.google.com/web/@32.74617752,129.31950128,6.83991611a,77186.95662495d,35y,0h,0t,0r

TableS 1-1 Results of S1-B sample long diameter (d), EDX analysis and materials

No, analy. spot	d (μm)	C (%)	O (%)	Al (%)	Si (%)	Cu (%)	Na (%)	Cl (%)	Others (%)	Materials
1, 1	2.8	74.6	2.9	0	0	0	12.1	10.3	0	A
1, 2	2.8	83.9	3.4	0	0	0	6.7	5.7	0.3[a]	A
1, 3	2.8	85.4	2.9	0	0	0	6.2	5.2	0.3[b]	A
1, 4	2.8	74.5	0	0	0	0	14.0	11.6	0	A
2, 1	0.7	86.2	13.1	0	0	0	0	0	0.7[c]	A
2, 2	0.7	85.0	15.0	0	0	50	50	50	0	A
3, 1	2.3	84.3	4.1	0	0	0	5.8	5.2	0.7[d]	A
3, 2	2.3	81.5	5.2	0	0	0	7.4	5.1	0.8[e]	A
3, 3	2.3	74.4	3.0	0	0	0	11.7	10.4	0.5[f]	A
4, 1	9.8	85.2	14.1	0	0	0	0	0.7	0	A
4, 2	9.8	81.6	17.7	0	0	0	0	0	0.7[g]	A
4, 3	9.8	85.3	11.7	0	0	0	1.0	1.3	0.7[h]	A
4, 4	9.8	81.3	15.4	0	0	0	1.3	1.0	0.9[i]	A
5, 1	2.0	68.8	26.2	0.5	3.8	0	0	0	0.7[j]	B
5, 2	2.0	31.1	54.3	0	13.2	0	0	0	0.6[k]	B
6, 1	5.0	59.7	33.4	0.4	5.8	0	0	0	0.7[l]	B
6, 2	5.0	26.4	55.8	0.8	15.8	0	1.2	0	0	B
6, 3	5.0	62.9	31.0	0.3	5.4	0	0	0	0.3[m]	B
7, 1	5.8	36.5	52.2	0	0	0	0	0	11.3[n]	A
7, 2	5.8	72.4	24.7	0	0	0	0	0	2.9[o]	A

% = mol%. A: Non Si polymers. B: Si paints. C: Cu paints. D: Other paints. E: Al coating laminate films. F: Shell.
[a)] Au = 0.3% [b)] Au = 0.3% [c)] Au = 0.7% [d)] Au = 0.7% [e)] Au = 0.8% [f)] Au = 0.5% [g)] Au = 0.5% [h)] Au = 0.7% [i)] Au = 0.9% [j)] Au = 0.7% [k)] Au = 0.6% [l)] Au = 0.7% [m)] Au = 0.3% [n)] Ca = 10.8%, Au = 0.5% [o)] Ca = 1.9%, Au = 1.0%

TableS 1-2 Results of S1-B sample long diameter (d), EDX analysis and materials

No, analy. spot	d (μm)	C (%)	O (%)	Al (%)	Si (%)	Cu (%)	Na (%)	Cl (%)	Others (%)	Materials
8, 1	0.9	17.7	56.3	6.1	14.1	0	5.8	0	0	B
9, 1	2.3	36.9	46.4	3.4	9.7	0	3.7	0	0	B
10, 1	3.3	66.2	25.4	1.7	4.2	0	1.3	0	1.2[a]	B
10, 2	3.3	21.3	55.2	4.6	13.6	0	4.6	0	0.9[b]	B
11, 1	2.5	81.3	17.6	0	0	0	0.6	0.6	0	A
11, 2	2.5	80.6	15.5	0	0	0	1.2	1.4	1.4[c]	A
11, 3	2.5	79.4	17.6	0	0	0	1.3	1	0.7[d]	A
12, 1	4.4	56.3	37.1	5.5	0	0	0	0.3	0.7[e]	E
12, 2	4.4	58.8	35.1	5.3	0	0	0	0	0.8[f]	E
12, 3	4.4	26.7	60.4	1.1	0	0	0.9	0	0.8[g]	E
12, 4	4.4	34.4	53.6	11.2	0	0	0	0	0.8[h]	E
13, 1	9.0	59.6	5.9	0	3.8	0	0	0	30.8[i]	B
13, 2	9.0	62.6	10.3	0	8.6	0	0	0	18.4[j]	B
14, 1	2.6	79.5	0	0	0	20.5	0	0	0	C
15, 1	9.0	38.0	8.4	0	0	53.7	0	0	0	C
16, 1	10.0	96.0	3.5	0	0	0	0	0	0.5[k]	A
17, 1	6.0	21.4	55.3	11.2	11.9	0	0	0	0	B
18. 1	10.0	11.4	62.4	12.7	13.6	0	0	0	0	B

% = mol%. A: Non Si polymers. B: Si paints. C: Cu paints. D: Other paints. E: Al coating laminate films. F: Shell.
[a] Mg= 0.4, Au = 0.3% [b] Au = 0.9% [c] Au = 1.4% [d] Au = 0.7% [e] Mo = 0.7% [f] S = 0.9% [g] Au = 0.8%
[h] Mo = 0.8% [i] Fe = 30.7% [j] Fe = 17.4%, Au = 1.0% [k] Au = 0.5%

TableS 1-3 Results of S1-B sample long diameter (d), EDX analysis and materials

No, analy. spot	d (μm)	C (%)	O (%)	Al (%)	Si (%)	Cu (%)	Na (%)	Cl (%)	Others (%)	Materials
19, 1	7.0	23.2	52.6	3.5	18.2	0	1.2	0	1.3[a]	B
20, 1	9.0	11.4	62.4	12.7	13.6	0	0	0	0	B
20, 2	9.0	12.0	60.8	13.3	13.9	0	0	0	0	B

% = mol%. A: Non Si polymers. B: Si paints. C: Cu paints. D: Other paints. E: Al coating laminate films. F: Shell.
a) K = 1.3%

TableS 1-4 Results of S1-B sample long diameter (d) and EDX analysis and materials

No, analy. spot	d (μm)	C (%)	O (%)	Al (%)	Si (%)	Cu (%)	Na (%)	Cl (%)	Others (%)	Materials
21, 1	28.0	73.4	21.6	0	0	1.1	1.7	1.4	0.8 [a]	C
21, 2	28.0	72.4	24.3	0	0.4	0.8	0	1.3	0.8[b]	C
22, 1	29.4	0	58.9	8.4	24.7	0	2.3	0	5.7[c]	B
22, 2	29.4	34.8	41.8	4.8	14.0	0	1.3	0	3.2[d]	B
23, 1	0.7	86.2	13.1	0	0	0	0	0	0.7	A
24, 1	5.5	62.2	29.4	0	0	0	0.7	0	7.7[e]	A*
25, 1	3.3	85.2	13.6	0	0.3	0	0	0.3	0	A
26, 1	21.4	84.4	15.1	0	0.2	0	0.3	0	0	A
27, 1	13.6	84.9	11.8	0	0.3	0	1.6	1.2	0.3[f]	A
28, 1	1.8	35.1	50.0	0	14.3	0	0.6	0	0	B
29, 1	4.3	43.7	43.2	0	11.8	0	0.9	0.2	0.2[g]	B
30, 1	5.2	89.4	7.7	0	0	0	0	0	2.9[h]	A
31, 1	9.8	40.6	50.0	0	0	0	0	0	9.5[i]	F
32, 1	5.1	50.6	37.3	2.5	6.7	0	0.5	0.4	2.1[j]	B
33, 1	1.6	85.3	9.1	0	0	0	3.3	2.3	0	A
34, 1	15.0	26.7	51.7	0	0	0	0	0	21.6[k]	F
35, 1	13.2	87.5	10.2	0	0.3	0	1.1	0.7	0.3[l]	A
36, 1	21.2	85.4	11.1	0	0	1.4	0	0	2.1[m]	C
37, 1	12.8	24.1	2.2	0	0	46.8	0	0	26.8[n]	C

% = mol%. A: Non Si polymers. B: Si paints. C: Cu paints. D: Other paints. E: Al coating laminate films. F: Shell.
*) MoS_2 lubricant contamination a) K = 0.5%, Mo = 0.3% b) K = 0.5%, S = 0.4% c) K = 0.7% d) K = 3.2% e) Mg = 0.2%, S = 3.6%, K = 0.4, Ca = 0.4, Cr = 0.3, Fe = 1.9, Mo = 1.0 f) K = 0.3% g) S = 0.2% h) K = 1.4%, S = 1.5% i) Ca = 9.5% j) K = 2.1% k) Mg = 9.8%, Ca = 11.9 l) K = 0.3% m) S = 1.4%, Ca = 0.7% n) Zn = 26.8%

TableS 1-5 Results of S1-B sample long diameter (d), EDX analysis and materials

No, analy. spot	d (μm)	C (%)	O (%)	Al (%)	Si (%)	Cu (%)	Na (%)	Cl (%)	Others (%)	Materials
38, 1	3.1	88.1	3.4	0	0	0	4.8	3.7	0	A
39, 1	2.0	87.1	2.7	0	0	0	5.7	4.5	0	A
40, 1	4.9	79.0	0	0	0	0	11.4	9.6	0	A
41, 1	4.9	26.0	2.0	0	0	45.3	0	0	26.7[a]	C
42, 1	3.0	77.3	3.7	0	0	11.9	0	0	7.2[b]	C
43, 1	26.9	77.5	16.4	0	0	0	4.2	1.8	0.2[c]	A
43, 2	26.9	76.1	15.2	0	0	0	5.0	3.0	0.5[d]	A

% = mol%. A: Non Si polymers. B: Si paints. C: Cu paints. D: Other paints. E: Al coating laminate films. F: Shell.
a) Zn = 26.7% b) Zn = 7.2% c) Mo = 0.2% d) S = 0.3%, K = 0.3%

TableS 2-1 Results of S1-D sample long diameter (d), EDX analysis and materials

No, analy. spot	d (μm)	C (%)	O (%)	Al (%)	Si (%)	Cu (%)	Na (%)	Cl (%)	Others (%)	Materials
1, 1	13.0	10.2	58.1	5.1	24.5	0	0	0	2.1[a]	B
2, 1	15.0	85.1	14.5	0	0	0	0	0.4	0	A
3, 1	18.0	99.0	0	0	0	0	0	1.0	0	A
4, 1	53.0	65.3	20.7	13.3	0.7	0	0	0	0	E
5, 1	24.0	64.5	29.5	0	0	0	1.5	2.8	1.7[b]	A
6, 1	10.0	77.0	18.0	0.5	1.6	0	0	2.0	0.9[c]	B
7, 1	14.0	50.2	44.5	0	5.4	0	0	0	0	B
8, 1	19.1	38.4	46.4	0	13.5	0	1.1	0.6	0	B
9, 1	21.8	81.1	13.8	0	0	0	2.7	1.5	0.9[d]	A
10, 1	10.0	60.2	34.0	0.6	3.2	0	0	0	2.1[e]	B
11, 1	40.0	74.6	21.5	0.5	0.8	0	0	0	2.5[f]	A
12, 1	5.3	61.6	32.7	1.1	1.5	0	0	0	3.2[g]	B
13, 1	13.3	66.2	20.6	2.4	4.2	0	0	0	6.7[h]	B
14, 1	6.0	54.5	33.1	5.4	5.7	0	0	0	1.4[i]	B
15, 1	12.3	62.8	30.3	0	1.5	0	0	0	5.5[j]	B
16, 1	17.3	69.1	28.4	0	0	0	1.4	1.1	0	A
16, 2	17.3	46.3	38.1	0	14.9	0	0	0	0.8[k]	--[l]

% = mol%. A: Non Si polymers. B: Si paints. C: Cu paints. D: Other paints. E: Al coating laminate films. F: Shell.
a) K = 1.4%, Au = 0.7% b) K = 1.7% c) Mg = 0.4%, Au = 0.5% d) K = 0.9% e) Ca = 1.2%, Au = 0.9% f) Ca = 1.5%, Au = 1.0% g) Ca = 2.1%, Au = 1.1% f) Ca = 5.1%, Au = 1.6% i) Ca = 1.4% j) Ca = 4.8%, Au = 0.7% k) Ca = 0.8% l) Diatom

TableS 2-2 Results of S1-D sample long diameter (d), EDX analysis and materials

No, analy. spot	d (μm)	C (%)	O (%)	Al (%)	Si (%)	Cu (%)	Na (%)	Cl (%)	Others (%)	Materials
17, 1	22.0	20.1	56.0	0.8	20.7	0	0.8	0	1.7[a]	B
17, 2	22.0	18.2	61.2	0	18.7	0	0.9	0.4	1.0[b]	B
17, 3	22.0	31.4	52.1	0	15.7	0	0.8	0	0	B
17, 4	22.0	14.5	31.6	0	52.3	0	0	0	1.6[c]	B
18 1	9.3	21.6	58.4	0	19.4	0	0.9	0	0	B
19, 1	22.0	44.6	42.9	2.0	9.5	0	0.6	0	0.4[d]	B
19, 2	22.0	76.8	12.6	1.0	9.2	0	0	0	0.5[e]	B
20, 1	1.4	56.9	35.3	1.2	5.3	0	0.6	0	0.6[f]	B
21, 1	40.0	75.8	20.5	1.0	2.7	0	0	0	0	B
22, 1	4.1	0	17.9	7.5	24.0	0	0	0	50.5[g]	B
23, 1	19.0	19.0	53.5	2.3	22.4	0	1.0	0	1.9[h]	B
24, 1	5.1	87.0	13.0	0	0	0	0	0	0	A
25, 1	34.0	45.6	44.0	0	0	0	0	0	10.4[i]	A(F)
25, 2	34.0	61.1	31.9	0	0.9	0	5.4	0	0.7[j]	A
26, 1	0.8	90.7	9.4	0	0	0	0	0	0	A
27, 1	5.8	73.9	16.4	0	0	9.7	0	0	0	C

% = mol%. A: Non Si polymers. B: Si paints. C: Cu paints. D: Other paints. E: Al coating laminate films. F: Shell.
a) Mg = 1.7% b) Au = 1.0% c) S = 1.6 d) Au = 0.4% e) Au = 0.5% f) Au = 0.6% g) Mg = 8.4%, Ca = 10.0%, Fe=28.9, Au = 3.3% h) Mg = 0.9%, Au = 0.9% i) Ca = 9.7%, Au = 0.7% j) Au = 0.7%

TableS 2-3 Results of S1-D sample long diameter (d), EDX analysis and materials

No, analy. spot	d (μm)	C (%)	O (%)	Al (%)	Si (%)	Cu (%)	Na (%)	Cl (%)	Others (%)	Materials
28, 1	4.3	45.8	2.2	0	0	34.0	0	0	18.0[a]	C
29, 1	10.2	29.9	2.3	0	0	43.8	0	0	24.0[b]	C
30, 1	7.5	16.1	2.2	0	0	51.7	0.8	0	28.8[c]	C
31, 1	6.0	0	0	0	0	64.9	0	0	35.1[d]	C
32, 1	5.7	45.1	2.4	0	0	33.1	0	0	19.4[e]	C
33, 1	8.6	31.6	1.8	0	0	43.8	0	0	22.8[f]	C
34, 1	8.0	15.3	3.8	1.9	0	47.4	0	0	31.7[g]	C
35, 1	1.4	26.1	0	0	0	48.9	0	0	25.0[h]	C
36, 1	57.5	75.4	15.9	1.3	4.0	0	1.0	1.5	0.9[i]	B
36, 2	57.5	82.3	13.3	0	0.2	0	1.3	2.2	0.6[j]	A
37, 1	11.4	89.8	9.9	0	0	0	0	0.3	0	A
37, 2	11.4	83.0	16.4	0	0	0	0.4	0.3	0	A
37, 3	11.4	87.4	11.3	0	0	0	0.7	0.6	0	A
38, 1	5.9	79.7	18.0	0	0	0	1.1	0.8	0.4[k]	A
39, 1	26.7	81.5	16.2	0	0	0	1.0	1.0	0.2[l]	A
39, 2	26.7	77.5	20.1	0	0	0	1.2	0.9	0.3[m]	A
40, 1	9.2	87.4	12.6	0	0	0	0	0	0	A

% = mol%. A: Non Si polymers. B: Si paints. C: Cu paints. D: Other paints. E: Al coating laminate films. F: Shell.
[a)] Zn = 18.0% [b)] Zn = 24.0% [c)] Zn = 28.8% [d)] Zn = 35.1% [e)] Zn = 19.4% [f)] Zn = 22.8% [g)] Zn = 31.2%, Pb = 0.5% [h)] Zn = 25.0% [i)] K = 0.9% [j)] Mg = 0.2%, Cr = 0.3 [k)] Cr = 0.4% [l)] Mg = 0.2% [m)] Mg = 0.2%

TableS 2-4 Results of S1-D sample long diameter (d), EDX analysis and materials

No, analy. spot	d (μm)	C (%)	O (%)	Al (%)	Si (%)	Cu (%)	Na (%)	Cl (%)	Others (%)	Materials
41, 1	0.9	71.2	25.7	1.0	1.3	0	0	0	0.8[a]	B
42, 1	3.7	81.1	18.5	0	0.4	0	0	0	0	A
43, 1	5.0	68.9	26.3	1.5	2.6	0	0	0	0.8[b]	B
44, 1	1.8	84.9	14.6	0	0.5	0	0	0	0	A
45 1	7.5	87.9	11.8	0	0.4	0	0	0	0	A
46, 1	6.2	51.4	40.2	0.4	7.4	0	0.6	0	0	B
47, 1	3.5	48.5	42.3	0.4	7.3	0	0	0.2	0	B
48, 1	5.8	55.6	34.6	2.0	3.5	0	0	0	4.4[c]	B
49, 1	8.7	63.5	30.2	1.1	2.3	0	0.4	0.3	2.3[d]	B
50, 1	6.0	47.5	38.9	1.5	12.2	0	0	0	0	B
51, 1	9.6	86.0	13.4	0	0.6	0	0	0	0	A
52, 1	4.0	84.7	15.3	0	0	0	0	0	0	A
53, 1	6.0	42.0	44.4	6.2	6.8	0	0	0	0.6[e]	B
54, 1	7.1	74.7	20.0	1.5	3.0	0	0.4	0.2	0.2[f]	B
55, 1	11.5	22.0	6.5	0	0	0	1.0	1.0	71.5[g]	D*
56, 1	30.7	72.4	13.6	0	1.1	0	3.0	5.6	4.3[h]	B
57, 1	12.3	52.2	27.9	1.4	6.4	0	3.0	2.0	7.1[i]	B

% = mol%. A: Non Si polymers. B: Si paints. C: Cu paints. D: Other paints. E: Al coating laminate films. F: Shell.
[*] MoS_2 lubricant contamination [a] Fe = 0.8% [b] Mg = 0.3%, K = 0.4% [c] Mg = 2.1%, K = 0.8%, Fe = 1.53% [d] Mg = 1.2%, K = 0.4%, Fe = 0.7% [e] Fe = 0.6% [f] K = 0.2% [g] S = 23.3%, K = 38.0%, Mo = 10.1 [h] S = 1.3%, K = 2.0%, Mg = 0.4, Ca = 0.5 [i] S = 0.9%, K = 1.2%, Mg – 1.8, Ca = 2.1, Fe = 1

TableS 2-5 Results of S1-D sample long diameter (d), EDX analysis and materials

No, analy. spot	d (μm)	C (%)	O (%)	Al (%)	Si (%)	Cu (%)	Na (%)	Cl (%)	Others (%)	Materials
58, 1	7.0	78.8	19.3	0	1.2	0	0.6	0.2	0	B
59, 1	7.1	80.2	18.9	0	0.7	0	0	0.2	0	A

% = mol%. A: Non Si polymers. B: Si paints. C: Cu paints. D: Other paints. E: Al coating laminate films. F: Shell.

TableS 3-1 Results of S3-B sample long diameter (d), EDX analysis and materials

No, analy. spot	d (μm)	C (%)	O (%)	Al (%)	Si (%)	Cu (%)	Na (%)	Cl (%)	Others (%)	Materials
1, 1	9.3	25.5	0	0	0	47.1	0	0	27.5[a]	C
2, 1	6.4	68.6	6.9	0	0	14.8	0	0	9.7[b]	C
3, 1	5.4	69.1	26.4	0.8	2.3	0	0.6	0.3	0.6[c]	B
4, 1	4.2	50.2	39.6	0	8.2	0	0.5	0.1	1.4[d]	B
5, 1	6.2	60.6	33.7	0	5.8	0	0	0	0	B
6, 1	2.0	86.6	10.5	0	0	0.89	0	0.9	1.1[e]	A
7, 1	10.0	0	0	0	0	64.9	0	0	35.1[f]	C
8, 1	2.5	87.8	11.9	0	0	0	0	0	0.3[g]	A
9, 1	75.0	72.3	20.8	0	0	0	3.2	3.3	0.4[h]	A
10, 1	100.0	71.7	22.3	0	0	0	2.9	3.2	0.5[i]	A
10, 2	100.0	87.4	12.6	0	0	0	0	0	0	A

% = mol%. A: Non Si polymers. B: Si paints. C: Cu paints. D: Other paints. E: Al coating laminate films. F: Shell.
[a)] Zn = 26.6%, Au = 0.9% [b)] Zn = 8.2%, Sb = 1.5% [c)] Fe = 0.6% [d)] Cr = 0.9%, Au = 0.5% [e)] K = 0.5%, Au = 0.6% [f)] Zn = 35.1% [g)] Au = 0.3% [h)] Mg = 0.4% [i)] Mg = 0.5%

TableS 3-2 Results of S3-B sample long diameter (d), EDX analysis and materials

No, analy. spot	d (μm)	C (%)	O (%)	Al (%)	Si (%)	Cu (%)	Na (%)	Cl (%)	Others (%)	Materials
11, 1	7.5	30.3	45.7	0	0	0	4.6	0.5	18.9[a]	D*
12, 1	5.0	41.0	45.1	0	0	0	3.1	0.3	10.5[b]	D*
13, 1	6.3	79.6	7.7	0	0	8.1	0	0	4.7[c]	C
14, 1	4.0	60.9	1.6	0	0	24.2	0	0	13.3[d]	C
15, 1	11.1	78.2	17.7	0	0	0	0.7	0	3.5[e]	A
15, 2	11.1	68.5	25.7	0	0	0	0.9	0	5.0[f]	A
16, 1	13.7	76.2	4.7	0	0	0	8.0	10.5	0.7[g]	A
16, 2	13.7	67.5	11.9	0	0	0	16.1	16.3	0	A
17, 1	1.8	77.8	19.9	0.2	0.9	0	0.4	0.3	0.6[h]	A
18, 1	3.0	57.6	37.0	0.2	4.6	0	0.5	0	0	B
19, 1	1.0	77.8	20.4	0	0	0	0.7	0	1.1[i]	A
20, 1	1.4	82.0	3.9	0	0	8.9	0	0	5.3[j]	C
21, 1	2.9	84.2	4.4	0	0	7.1	0	0	4.3[k]	C
22, 1	5.0	39.1	0	0	0	35.7	0	0	25.2[l]	C
23, 1	6.9	49.8	4.1	0	0	29.8	0	0	16.3[m]	C
24.1	63.9	65.6	4.5	0	0	18.7	0	0	11.3[n]	C
24.2	63.9	79.1	20.1	0	0	0	0	0.2	0.6[o]	--

% = mol%. A: Non Si polymers. B: Si paints. C: Cu paints. D: Other paints. E: Al coating laminate films. F: Shell.
*) MoS_2 lubricant contamination a) S = 8.4%, K = 0.7%, Ca = 8.1, Mo = 1.7% b) S = 4.5%, K = 0.4%, Ca = 4.6, Mo = 1.1% c) Ca = 0.3%, Zn = 4.4% d) Zn = 12.9%, Au = 0.4% e) S = 1.7%, Ca = 1.8% f) S = 2.6%, Ca = 2.4% g) S = 0.3%, Ca = 0.3% h) Ti = 0.2%, Au = 0.3% i) S = 0.6%, Ca = 0.4% j) Zn = 5.3% k) Zn = 4.3% l) Zn = 25.2% m) Zn = 16.3% n) Zn = 10.8%, Au = 0.4% o) Ca = 0.4%, Au = 0.2%

TableS 3-3 Results of S3-B sample long diameter (d), EDX analysis and materials

No, analy. spot	d (μm)	C (%)	O (%)	Al (%)	Si (%)	Cu (%)	Na (%)	Cl (%)	Others (%)	Materials
25, 1	11.4	74.5	14.9	0	0.3	0	2.5	4.0	3.8[a]	A
26, 1	5.0	78.7	18.3	0.3	0.7	0	0.9	0.3	0.9[b]	A
27, 1	5.7	85.5	12.0	0	0.3	8.1	0.8	0.5	1.0[c]	C
28, 1	12.5	23.9	0	0	0	49.4	0	0	26.7[d]	C
29, 1	1.2	75.3	3.1	0	0	13.8	0	0	7.8[e]	C
30, 1	14.0	33.9	0	0	0	0	0.2	0	65.9[f]	A
31, 1	11.0	49.3	0	0	0	0	0	0	50.8[g]	A
32, 1	2.9	85.6	3.4	0	0	6.8	0	0	4.3[h]	C
33, 1	8.6	20.9	1.6	0	0	49.4	0	0	28.1[i]	C
34, 1	53.8	81.6	18.1	0	0	0	0	0	0.3[j]	A
34, 2	53.8	85.3	14.7	0	0	0	0	0	0	A
35, 1	10.8	78.6	21.4	0	0	0	0	0	0	A
36, 1	21.0	58.9	13.8	0	0	0	14.3	11.2	1.8[k]	A
36, 2	21.0	66.2	3.7	0	0	0	14.1	16.0	0	A
37, 1	5.6	60.3	0	0	0	0	8.7	30.8	0.3[l]	A
38.1	18.8	0	0	0	0	0	48.4	51.6	0	--*
39.1	8.8	0	0	0	0	0	47.9	52.1	0	--*
40.1	14.1	35.4	0	0	0	0	0.5	0.3	63.8[m]	A

% = mol%. A: Non Si polymers. B: Si paints. C: Cu paints. D: Other paints. E: Al coating laminate films. F: Shell. *) NaCl crystal a) K = 3.8% b) K = 0.5%, Au = 0.4% c) Ca = 0.8%, Au = 0.2% d) Zn = 25.9%, Au = 0.8% e) Zn = 7.8% f) F = 65.7%, Au = 0.2% g) F = 50.8% h) Zn = 4.1%, Au = 0.2% i) Zn = 26.6%, Pb = 1.6% j) Zn = 5.3% k) S = 0.9%, Ca = 0.9% l) Au = 0.3% m) F = 62.5%, Au = 1.3%

TableS 3-4 Results of S3-B sample long diameter (d), EDX analysis and materials

No, analy. spot	d (μm)	C (%)	O (%)	Al (%)	Si (%)	Cu (%)	Na (%)	Cl (%)	Others (%)	Materials
41, 1	7.9	13.5	0	0	0	52.7	0	1.0	32.8[a]	C
42, 1	44.7	0	7.2	0	0	0	46.0	46.9	0	--*
43, 1	7.8	50.2	39.6	0	8.2	0	0.5	0.1	1.4[b]	B
44, 1	6.5	60.6	33.7	0	5.8	0	0	0	0	B

% = mol%. A: Non Si polymers. B: Si paints. C: Cu paints. D: Other paints. E: Al coating laminate films. F: Shell.
*) NaCl crystal a) Zn = 31.5%, Au = 1.3% b) Cr = 0.9%, Au = 0.5%

TableS 4-1 Results of S3-D sample long diameter (d), EDX analysis and materials

No, analy. spot	d (μm)	C (%)	O (%)	Al (%)	Si (%)	Cu (%)	Na (%)	Cl (%)	Others (%)	Materials
1, 1	6.0	25.1	53.3	0	12.2	0	0	0	9.5[a]	B
2, 1	12.0	0	55.6	0	27.8	0	0	0	16.6[b]	B
3, 1	3.6	36.0	43.2	0	12.0	0	0	0.1	8.7[c]	B
4, 1	31.0	77.5	9.1	0	0	9.2	0	0	4.3[d]	C
5, 1	5.0	36.5	9.0	0	0	35.6	0	0	18.9[e]	C
6, 1	20.0	68.8	9.3	0	0	13.3	0	1.2	7.5[f]	C
7, 1	15.0	0	57.8	0	0	26.3	0	0	15.9[g]	D
7, 2	15.0	0	62.4	0	0	22.0	0	0	15.6[h]	D
8, 1	1.8	60.2	0	0	0	7.2	0	0	32.6[i]	C
9, 1	2.0	0	0	0	0	100	0	0	0	C
10, 1	12.5	76.0	13.8	0	0	0	0	0	10.2[j]	A*
10, 2	12.5	65.4	17.6	0	0	0	0	0	17.1[k]	A*
11, 1	14.2	0	66.3	0	19.3	0	0	0	19.3[l]	B
11, 2	14.2	10.7	61.9	0	14.9	0	0	0	12.4[m]	B
11, 3	14.2	35.2	50.9	0	7.7	0	0	0	6.3[n]	B
12, 1	37.3	83.0	16.8	0	0	0	0	0	0.2[o]	A
12, 2	37.3	82.4	17.2	0	0	0	0.4	0	0	A

% = mol%. A: Non Si polymers. B: Si paints. C: Cu paints. D: Other paints. E: Al coating laminate films. F: Shell. [*)] MoS_2 lubricant contamination [a)] Mg = 9.5% [b)] Mg = 16.6% [c)] Mg = 8.4%, Au = 0.4% [d)] Zn = 4.3% [e)] Zn = 18.9% [f)] Zn = 6.7%, Au = 0.8% [g)] Mg = 15.9% [h)] Mg = 15.6% [i)] Au = 32.6% [j)] S = 3.4%, K = 5.1%, Mo = 1.7% [k)] S = 5.9%, K = 8.8%, Mo = 2.4% [l)] Mg = 19.3% [m)] Mg = 11.9%, Au = 0.5% [n)] Mg = 6.3% [o)] Au = 0.2%

TableS 4-2 Results of S3-D sample long diameter (d), EDX analysis and materials

No, analy. spot	d (μm)	C (%)	O (%)	Al (%)	Si (%)	Cu (%)	Na (%)	Cl (%)	Others (%)	Materials
13, 1	7.1	49.1	34.2	0	9.9	0	0	0	6.9[a]	B
13, 2	7.1	26.0	55.0	0	10.4	0	0	0	8.6[b]	B
14, 1	15.0	29.5	48.0	0	12.7	0	0	0	9.8[c]	B
14, 2	15.0	32.3	46.2	0	12.5	0	0	0	9.1[d]	B
15, 1	3.8	34.3	46.9	0	18.8	0	0	0	0	B
16, 1	28.5	79.4	18.6	0	0	0	0	0.6	1.3[e]	A
16, 2	28.5	76.2	23.8	0	0	0	0	0	0	A
17, 1	8.2	72.3	19.0	0	0.9	0	0	0	7.7[f]	B
17, 2	8.2	61.1	17.5	0.4	20.6	0	0	0	0.4[g]	B
18, 1	15.6	83.3	15.8	0	0	0	0	0.2	0.7[h]	A
19, 1	8.5	75.4	24.3	0	0	0	0	0.3	0	A
19, 2	8.5	74.6	24.7	0	0	0	0	0.7	0	A
20, 1	11.1	12.7	60.0	0	15.5	0	0	0	11.9[i]	B
21 1	2.9	0	0	0	0	100	0	0	12.4	C
22, 1	1.5	59.7	33.3	0.2	5.9	0	0	0	0.9[j]	B
23, 1	1.1	59.7	33.3	0.2	5.9	0	0	0	0.9[k]	B

% = mol%. A: Non Si polymers. B: Si paints. C: Cu paints. D: Other paints. E: Al coating laminate films. F: Shell. [*] MoS_2 lubricant contamination [a] Mg = 6.9% [b] Mg = 8.6% [c] Mg = 9.6%, Au = 0.3% [d] Mg = 9.1% [e] S = 0.7%, Au = 0.6% [f] Ca = 7.7% [g] Mg = 0.4% [h] Mg = 0.3%, S = 0.5% [i] Mg = 11.9% [j] Ti = 0.7%, Au = 0.2% [k] Ti = 0.7%, Au = 0.2%

TableS 5-1 Results of S5-B sample long diameter (d), EDX analysis and materials

No, analy. spot	d (μm)	C (%)	O (%)	Al (%)	Si (%)	Cu (%)	Na (%)	Cl (%)	Others (%)	Materials
1, 1	22.0	81.6	14.1	0.4	1.0	0	0.3	2.5	0.2[a]	A
2, 1	2.5	69.9	5.2	0	0	15.5	0	0	9.4[b]	C
3, 1	23.8	23.8	0	0	0	49.2	0	0	27.1[c]	C
3, 2	23.8	30.6	2.6	0	0	43.3	0	0	23.6[d]	C
4, 1	32.7	86.2	13.1	0	0	0	0	0	0	A
5, 1	4.2	66.9	23.4	0	0	0	1.0	0	8.7[e]	A*
6, 1	15.0	87.1	12.6	0	0	0	0.3	0	0	A
6, 2	15.0	83.3	11.4	1.6	2.0	0	0.8	0.2	0.8[f]	--
7, 1	1.1	86.3	10.9	0	0	0	1.1	0.2	1.5[g]	A
8, 1	20.0	0	0	0	0	63.1	0	0	36.9[h]	C
9, 1	2.0	69.5	24.2	0	0	1.9	0	0.8	3.6[i]	C
10, 1	3.0	79.5	5.1	0	0	9.5	0	0	5.8[j]	C
11, 1	4.2	34.7	0	0	0	28.8	0	0	36.5[k]	C
12, 1	2.5	39.6	4.0	0	0	35.1	0	0	21.3[l]	C
13, 1	6.9	34.5	3.3	0	0	35.7	0	0	26.5[m]	C
14, 1	2.8	76.5	5.5	0	0	11.3	0	0	6.72[n]	C

% = mol%. A: Non Si polymers. B: Si paints. C: Cu paints. D: Other paints. E: Al coating laminate films. F: Shell. [*)] MoS_2 lubricant contamination [a)] Mg = 0.2% [b)] Zn = 9.1%, Pb = 0.4% [c)] Zn = 27.1% [d)] Zn = 23.6% [e)] S = 3.3%, K = 4.2%, Mo = 1.2% [f)] K = 0.8% [g)] S = 0.6%, K = 1.0% [h)] Zn = 36.9% [i)] S = 1.1%, K = 0.8%, Fe = 1.7% [j)] Zn = 5.6%, Pb = 0.2% [k)] F = 7.7%, P = 3.1%, Fe = 8.3%, Zn = 17.4% [l)] Zn = 21.3% [m)] F = 1.5%, Fe = 3.6%, Zn = 21.3% [n)] Zn = 6.3%, Pb = 0.4%

TableS 5-2 Results of S5-B sample long diameter (d), EDX analysis and materials

No, analy. spot	d (μm)	C (%)	O (%)	Al (%)	Si (%)	Cu (%)	Na (%)	Cl (%)	Others (%)	Materials
15, 1	23.5	0	4.9	0	0	58.4	0	0	36.8[a]	C
16, 1	3.0	41.4	6.9	0	0	31.9	0	0	19.8[b]	C
17, 1	6.8	13.4	68.1	0	0	0	0	0	18.5[c]	D
18, 1	23.3	81.6	16.1	0	0	2.4[d]	0	0	0	C
19, 1	2.0	82.1	17.8	0	0	0	0	0	0	A
20, 1	6.3	62.3	20.6	0	0.2	0	1.0	0	16.8[e]	A
21, 1	10.0	24.9	49.2	0	0	0	0.3	0	25.9[f]	F*
22, 1	100.0	86.0	11.9	0	0	1.9	0	0	0.2[g]	C
23, 1	3.6	76.5	2.5	0	0	13.9	0	0	7.1[h]	C
24, 1	54.5	78.0	13.4	0	1.2	0	0	0.7	6.8[i]	E
24, 2	54.5	80.8	15.8	0	0	0	0	0.3	3.6[j]	E
25, 1	8.7	74.3	23.4	0	1.3	0	0.3	0.2	1.2[k]	A
25, 2	8.7	83.3	10.6	0	0.9	0.9	0.3	0.4	3.7[l]	A
26, 1	23.3	79.8	18.5	0	0.5	35.1	0.4	0.4	0.4[m]	C
27, 1	11.7	44.8	38.9	0	9.5	0	0	0	7.0[n]	B
27, 2	11.7	43.5	40.4	0	9.2	0	0	0	6.9[o]	B

% = mol%. A: Non Si polymers. B: Si paints. C: Cu paints. D: Other paints. E: Al coating laminate films. F: Shell.
*) MoS_2 lubricant contamination a) Zn = 33.5%, Pb = 3.3% b) Zn = 18.5%, Pb = 1.3% c) S = 6.3%, K = 2.8%, Fe = 8.7%, Tl = 0.8% d) Cu containation e) S = 6.0%, Ca = 9.0%, Mo = 1.8% f) S = 9.7%, Ca = 12.9%, Mo = 3.4% g) S = 0.6%, K = 1.0% h) Zn = 7.1% i) P = 2.8%, S = 0.9%, K = 3.1% j) P = 1.5%, S = 0.4%, K = 1.3% k) P = 0.6%, S = 0.2%, K = 0.4% l) P = 1.7%, K = 1.5%, Nb = 0.5% m) Ca = 0.4% n) Mg = 7.0% o) Mg = 6.9%

TableS 5-3 Results of S5-B sample long diameter (d), EDX analysis and materials

No, analy. spot	d (μm)	C (%)	O (%)	Al (%)	Si (%)	Cu (%)	Na (%)	Cl (%)	Others (%)	Materials
28, 1	26.7	81.8	17.6	0	0	0	0.4	0.3	0	A
29, 1	50.0	80.6	16.8	0	0.3	0	0.8	0.7	0.8[a]	A
29, 2	50.0	82.8	16.4	0	0	0	0.4	0.2	0.2[b]	A

% = mol%. A: Non Si polymers. B: Si paints. C: Cu paints. D: Other paints. E: Al coating laminate films. F: Shell.
[a)] S = 0.2%, K = 0.5% [b)] K = 0.2% [c)] Mg = 7.0%

TableS 5-4 Results of S5-B sample long diameter (d), EDX analysis and materials

No, analy. spot	d (μm)	C (%)	O (%)	Al (%)	Si (%)	Cu (%)	Na (%)	Cl (%)	Others (%)	Materials
30, 1	7.4	81.1	15.2	0	0.2	0	1.4	0.9	1.2[a]	A
31, 1	30.4	81.5	18.5	0	0	0	0	0	0	A
32, 1	8.0	94.2	3.5	0	0	2.3	0	0	0	C
33, 1	4.2	75.0	1.7	0	0	13.1	0	0	10.2[b]	C
34, 1	26.4	79.2	19.1	0	0	0	0.8	0.5	0.4[c]	A
34, 2	26.4	75.3	22.9	0	0	0	0.9	0.5	0.4[d]	A
35, 1	18.3	73.3	0	0	0.9	0	0.8	9.8	15.2[e]	A
35, 2	18.3	74.9	14.6	0	0	0	0.7	4.8	5.1[f]	A
36, 1	24.0	73.9	7.2	0	0.3	0	0.8	8.3	9.5[g]	A
37, 1	17.3	55.8	33.3	1.4	8.7	0	0	0.2	0.7[h]	B
37, 2	17.3	62.1	17.6	1.6	16.4	0	0	0	1.9[i]	B
38, 1	9.0	32.9	40.9	3.8	20.4	0	1.0	0	1.1[j]	B
39, 1	20.0	53.1	2.9	0	0	28.0	0	0	16.0[k]	C
39, 2	20.0	65.2	3.7	0	0	19.0	0	0.4	12.1[l]	C
40, 1	10.0	79.5	5.5	0	0	0	1.8	13.0	0.1[m]	A
40, 2	10.0	46.6	0	0	0	0	24.4	29.0	0	A

% = mol%. A: Non Si polymers. B: Si paints. C: Cu paints. D: Other paints. E: Al coating laminate films. F: Shell.
*) MoS_2 lubricant contamination a) K = 0.8%, Ca = 0.4% b) Zn = 9.8%, Au = 0.4% c) K = 0.4% d) K = 0.4% e) S = 6.0%, Ca = 9.0%, Mo = 1.8% f) K = 4.7%, Au = 0.4% g) K = 9.5% h) Mg = 0.3%, K = 0.3% i) K = 0.6%, Fe = 0.9%, Au = 0.4% j) K = 1.1% k) Zn = 15.3%, Pb = 0.8% l) K = 0.2%, Zn = 11.9% m) Au = 0.1%

TableS 5-5 Results of S5-B sample long diameter (d), EDX analysis and materials

No, analy. spot	d (μm)	C (%)	O (%)	Al (%)	Si (%)	Cu (%)	Na (%)	Cl (%)	Others (%)	Materials
41, 1	16.7	25.9	59.2	0.6	13.0	0	1.1	0	0.3[a]	B
41, 2	16.7	9.6	57.1	1.1	30.1	0	1.1	0	1.1[b]	B
42, 1	20.7	92.1	7.9	0	0	0	0	0	0	A
43, 1	8.0	79.1	15.2	0	0	0	0.7	2.6	2.4[c]	A
43, 2	15.5	75.8	16.1	0	0	0	1.1	4.1	2.9[d]	A
44, 1	15.0	80.1	19.9	0	0	0	0	0	0	A
44, 2	15.0	81.8	18.2	0	0	0	0	0	0	A
45, 1	23.3	88.1	2.2	0	0	5.8	0	0	4.0[e]	C
45, 2	23.3	89.8	1.7	0	0	4.3	0.8	0.2	4.0[f]	C
46, 1	18.8	45.3	2.7	0	0	33.5	0	0	18.5[g]	C
46, 2	18.8	34.2	1.5	0	0	41.2	0	0	23.2[h]	C
47, 1	5.0	32.5	48.3	0	10.9	0	0	0	8.3[i]	B
48, 1	5.4	16.9	55.6	0	15.7	0	0	0	11.8[j]	B
49, 1	21.0	85.0	15.0	0	0	0	0	0	0	A
50, 1	2.0	81.5	18.3	0	0	0	0	0	0.2	A
51, 1	21.7	94.9	5.1	0	0	0	0	0	0	A
51, 2	21.7	94.4	5.6	0	0	0	0	0	0	A
52, 1	12.5	26.1	44.8	0	17.4	0	0	0	11.8[k]	B
52, 2	12.5	0	63.3	0	21.3	0	0	0	15.4[l]	B

% = mol%. A: Non Si polymers. B: Si paints. C: Cu paints. D: Other paints. E: Al coating laminate films. F: Shell.
a) Au = 0.3% b) K = 0.5%, Au = 0.6% c) S =0.2%, K = 0.4%, Ca = 1.8% d) Ca = 2.9% e) Zn = 3.9%, Au = 0.2% f) S = 0.5%, K = 0.9%, Zn = 2.6% g) Zn = 17.9%, Au = 0.6% h) Zn = 22.9%, Au = 0.3% i) K = 0.6%, Fe = 0.9%, Au = 0.4% j) Mg = 8.3% k) Mg = 11.8% l) Mg = 15.4+%

TableS 6-1 Results of S5-D sample long diameter (d), EDX analysis and materials

No, analy. spot	d (μm)	C (%)	O (%)	Al (%)	Si (%)	Cu (%)	Na (%)	Cl (%)	Others (%)	Materials
1, 1	22.0	60.0	31.3	2.5	2.0	0	0.8	1.4	2.0[a]	B
2, 1	19.0	85.1	12.7	0.7	0	0	0.4	0.7	0.5[b]	A
3, 1	5.5	31.2	53.9	0	0	0	0	0	14.9[c]	F
4, 1	15.0	65.7	6.4	0.9	25.8	0	0	0	1.2[d]	B
4, 2	15.0	45.6	36.6	0.7	15.8	0	0.9	0.4	0	B
5, 1	12.6	78.4	13.8	0	0	0	3.9	3.7	0.3[e]	A
6, 1	2.0	79.3	11.8	0	0	0	4.7	3.9	0.4[f]	A
7, 1	23.5	53.1	37.4	0.4	8.4	0	0.5	0	0.2[g]	B
7, 2	23.5	0	68.8	1.3	28.2	0	1.6	0	0	B
8, 1	61.5	27.4	44.7	1.3	25.9	0	0.6	0	0	B
8, 2	61.5	0	63.6	1.5	33.6	0	1.3	0	0	B
9, 1	26.9	72.9	22.1	0	0	0	2.1	2.3	0.6[h]	A
10, 1	30.7	73.4	20.3	0	0	0	3.0	2.7	0.7[i]	A
11, 1	8.0	77.6	19.5	0	0	0	1.4	1.2	0.3[j]	A
11, 2	8.0	78.0	20.0	0	0	0	1.2	0.9	0	A
12, 1	1.5	76.2	15.1	0	0	0	4.4	3.8	0.4[k]	A
13, 1	4.7	65.3	7.8	0	0	0	14.2	12.7	0	A
14, 1	5.0	77.2	6.9	0	0	0	7.1	8.1	0.6[l]	A

% = mol%. A: Non Si polymers. B: Si paints. C: Cu paints. D: Other paints. E: Al coating laminate films. F: Shell.
a) Mg = 0.3%, K = 0.6%, Ca = 0.4%, Mo = 0.6% b) S = 0.2%, Ca = 0.3% c) Ca = 14.9% d) K = 0.6%, Au = 0.6% e) Mg = 0.3% f) Mg = 0.4% g) Au = 0.2% h) Mg = 0.6% i) Mg = 0.7% j) Mg = 0.2% k) Mg = 0.4% l) Zn = 0.2%, Mg = 0.4%

TableS 6-2 Results of S5-D sample long diameter (d), EDX analysis and materials

No, analy. spot	d (μm)	C (%)	O (%)	Al (%)	Si (%)	Cu (%)	Na (%)	Cl (%)	Others (%)	Materials
15, 1	40.9	85.1	14.8	0	0	0	0	0	0.2[a]	A
15, 2	40.9	88.8	10.9	0	0	0	0	0.2	0	A
16, 1	6.8	39.0	44.5	0	8.1	0	1.1	0.8	6.6[b]	B
16, 2	6.8	55.0	32.6	0	7.0	0	0	0	5.4[c]	B
17, 1	35.3	91.5	8.3	0	0	0	0	0.3	0	A
17, 2	35.3	84.5	13.9	0	0.3	0	0.	0.5	0.3[d]	A
18, 1	5.8	17.4	0	0	0	53.0	0	0	29.7[e]	C
18, 2	5.8	36.5	3.1	0	0	39.4	0	0	21.0[f]	C
19, 1	3.5	34.0	1.6	0	0	39.4	0	0	25.0[g]	C
20, 1	10.0	11.7	62.4	1.0	23.2	0	0.6	0	0	B
20, 2	10.0	52.9	37.0	0.4	8.8	0	0.8	0	0	B
21, 1	16.0	62.5	33.8	0	3.7	0	0	0	0	B
22, 1	10.7	74.7	15.3	0	0	0	5.5	4.2	0.4[h]	A
22, 2	10.7	79.4	14.5	0	0	0	2.8	2.9	0.3[i]	A
23, 1	4.3	47.4	7.5	0	0	28.5	0	0	16.5[j]	C
24, 1	8.3	14.1	0	0	0	55.8	0	0	30.1[k]	C
25, 1	5.7	25.7	0	0	0	43.3	0	0	31.1[l]	C
26, 1	5.6	58.5	0	0	0	27.1	0	0	14.4[m]	C
27, 1	2.9	60.8	6.9	0	0.6	19.5	0	0	12.2[n]	C

% = mol%. A: Non Si polymers. B: Si paints. C: Cu paints. D: Other paints. E: Al coating laminate films. F: Shell.
a) Au = 0.2% b) Mg = 6.6% c) Mg = 5.4% d) K = 0.3% e) Zn = 29.7% f) Zn = 21.0% g) Zn = 23.6%, Pb = 0.7%, Au = 0.7% h) Mg = 0.4% i) Mg = 0.3% j) Zn = 16.5% k) Zn = 30.1% l) Zn = 31.1% m) Zn = 14.4%

TableS 6-3 Results of S5-D sample long diameter (d), EDX analysis and materials

No, analy. spot	d (μm)	C (%)	O (%)	Al (%)	Si (%)	Cu (%)	Na (%)	Cl (%)	Others (%)	Materials
28, 1	3.3	55.4	5.9	0	0	17.7	0	0	21.1[a]	C
29, 1	8.5	16.3	57.1	0	15.1	0	0	0	15.5[b]	B
29, 2	8.5	8.2	59.8	0	18.2	0	0	0	13.8[c]	B
30, 1	1.9	67.5	2.6	0	0	19.0	0	0	11.0[d]	C
31, 1	3.5	36.3	3.1	0	0	38.8	0	0	21.8[e]	C
32, 1	5.0	73.2	2.5	0	0	14.9	0.	0	9.4[f]	C
33, 1	4.0	86.5	6.5	0	0	4.5	0	0	2.5[g]	C
34, 1	16.0	87.3	8.4	0.6	1.8	0	0	1.2	0.8[h]	B
34, 2	16.0	77.8	19.0	0.3	1.0	39.4	0.7	0.7	0.5[i]	B
35, 1	9.0	71.8	4.4	0	0	0	13.1	10.6	0	A
35, 2	9.0	67.2	8.7	0	0	0	13.0	10.6	0.5[j]	A
36, 1	8.0	17.3	56.9	0	14.3	0	0	0	11.5[k]	B
37, 1	7.0	50.8	38.7	0	4.7	0	0	0	4.8[l]	B
38, 1	4.0	42.0	43.2	0	8.1	0	0	0	6.6[m]	B
39, 1	14.3	78.0	17.2	0	0.3	0	1.1	1.8	1.6[n]	A
40, 1	30.0	70.2	20.5	0	0	0	6.2	2.9	0.3[o]	A
40, 2	30.0	71.5	19.5	0	0	0	2.7	3.4	3.0[p]	A
41, 1	9.3	61.2	6.3	0	0	0	14.6	17.6	0.4[q]	A
41, 2	9.3	37.7	4.1	0	0	0	30.4	27.9	0	A

% = mol%. A: Non Si polymers. B: Si paints. C: Cu paints. D: Other paints. E: Al coating laminate films. F: Shell.
[a)] S = 4.4%, Zn = 10.3%, Pb = 6.4% [b)] Mg = 6.6% [c)] Mg = 5.4% [d)] Zn = 11.0% [e)] Zn = 21.8% [f)] Zn = 9.1%, Au = 0.3% [g)] Zn = 2.5% [h)] Mg = 0.4%, S = 0.2%, Ca = 0.2% [i)] Mg = 0.5% [j)] K = 0.5% [k)] Mg = 11.5% [l)] Mg = 4.7%, Au = 0.2% [m)] Mg = 6.6%, [n)] Zn = 1.6% [o)] K = 0.3% [p)] K = 2.7%, Mo = 0.2% [q)] K = 0.4%

TableS 6-4 Results of S5-D sample long diameter (d), EDX analysis and materials

No, analy. spot	d (μm)	C (%)	O (%)	Al (%)	Si (%)	Cu (%)	Na (%)	Cl (%)	Others (%)	Materials
42, 1	3.8	51.7	33.9	0	0	0	0	0.7	13.7[a]	A
43, 1	28.5	70.7	22.2	0	0.2	0	1.3	2.4	3.3[b]	A
43, 2	28.5	75.0	23.6	0	0	0	0.7	0.4	0.3[c]	A
44, 1	3.6	42.6	1.9	0	0	35.2	0	0	20.3[d]	C
45, 1	7.1	17.8	0	0	0	52.3	0	0	29.9[e]	C

% = mol%. A: Non Si polymers. B: Si paints. C: Cu paints. D: Other paints. E: Al coating laminate films. F: Shell.
a) Mg = 5.7%, S = 7.6%, TI = 0.4% b)K = 3.1%, Au = 0.2% c)K = 0.3% d)Zn = 19.7%, Au = 0.6% e)Zn = 29.9%

TableS 7-1 Results of S7-B sample long diameter (d), EDX analysis and materials

No, analy. spot	d (μm)	C (%)	O (%)	Al (%)	Si (%)	Cu (%)	Na (%)	Cl (%)	Others (%)	Materials
1, 1	30.0	72.9	23.9	0	0	0	1.7	1.2	0.4[a]	A
2, 1	15.9	84.5	13.1	0	0	15.5	0	0	2.4[b]	A
3, 1	7.7	77.8	14.6	0	0	0	0	0	7.5[c]	A*
3, 2	7.7	77.5	15.0	0	0.3	0	0	0	7.3[d]	A*
4, 1	5.5	19.0	2.0	0	0	50.9	0	0	28.1[e]	C
5, 1	4.6	33.0	0	0	0	43.0	0	0	24.0[f]	C
6, 1	3.4	69.8	2.7	0	0	17.1	0	0	10.4[g]	C
7, 1	27.3	82.5	13.6	0	0	0	1.6	1.3	1.1[h]	A
7, 2	27.3	78.2	20.1	0	0	0	0.7	0.7	0.4[i]	A
8, 1	29.4	75.4	21.5	0	0	0	1.4	0.9	0.8[j]	A
9, 1	15.0	89.3	10.7	0	0	0	0	0	0	A
9, 2	15.0	89.1	10.9	0	0	0	0	0	0	A
10, 1	5.0	73.8	22.5	0	2.0	0	0	0	1.8[k]	B
10, 2	5.0	70.2	25.9	0	2.0	0	0	0	1.9[l]	B
10, 3	5.0	70.6	26.0	0	2.0	0	0	0	1.5[m]	B

% = mol%. A: Non Si polymers. B: Si paints. C: Cu paints. D: Other paints. E: Al coating laminate films. F: Shell. *) MoS_2 lubricant contamination a) K = 0.2% b) Sb = 2.4% c) S = 4.0%, Ti = 0.6%, Cr =0.7, Mo = 2.1% d) S = 3.5%, Ti = 1.1%, Cr =0.5, Mo = 2.1% e) Zn = 28.1% f) Zn = 24.0% g) Zn = 10.4% h) S = 0.3%, K = 0.8% i) S = 1.1%, K = 0.8%, Fe = 1.7% j) Zn = 5.6%, Pb = 0.2% k) Mg = 1.4%, Au = 0.3% l) Mg = 1.6%, Au = 0.3% m) Mg = 1.5%

TableS 7-2 Results of S7-B sample long diameter (d), EDX analysis and materials

No, analy. spot	d (μm)	C (%)	O (%)	Al (%)	Si (%)	Cu (%)	Na (%)	Cl (%)	Others (%)	Materials
11, 1	33.3	78.6	18.8	0	0	0	1.1	1.0	0.6[a]	A
11, 2	33.3	86.2	7.9	0	0	0	1.6	2.0	2.4[b]	A
12, 1	9.4	0	0	0	0	61.4	0	0	38.6[c]	C
12, 2	9.4	33.2	0	0	0	43.8	0	0	23.0[d]	C
13, 1	3.8	76.1	21.3	0	0	0	1.3	1.1	0.2[e]	A
14, 1	27.6	76.9	21.0	0	0	0	1.0	1.1	0	A
14, 2	27.6	76.3	21.2	0	0	0	1.2	1.1	0.3[f]	A
15, 1	7.6	84.0	10.9	0	0	3.3*	0	0	1.8[g]	A
15, 2	7.6	80.8	19.2	0	0	0	0	0	0	A
15, 3	7.6	84.9	15.1	0	0	0	0	0	0	A
16, 1	14.4	54.8	31.1	0	13.0	0	0.3	0	0.8[h]	B
16, 2	14.4	49.8	38.4	0	11.4	0	0	0	0.5[i]	B
17, 1	7.3	29.8	0	0	0	43.9	0	0	26.3[j]	C
18, 1	8.3	68.0	0	0	0	32.0	0	0	0	C
19, 1	3.0	69.9	3.0	0	0	17.4	0	0	9.8[k]	C
20, 1	55.6	39.2	1.8	5.2	0	33.5	0	0	20.4[l]	C
21, 1	55.6	0	54.3	58.7	0	0	0	0	0	D*
22, 1	10.0	10.7	52.8	0	23.1	0	0	0	13.3[m]	B
22, 2	10.0	30.2	47.6	0	12.9	0	0	0	9.3[n]	B

% = mol%. A: Non Si polymers. B: Si paints. C: Cu paints. D: Other paints. E: Al coating laminate films. F: Shell.
*) Al_2O_3 contamination a) K = 0.6% b) K= 1.5%, Mo =0.6%, Au = 0.4 c) Zn = 36.8%, Au= 1.8% d) Zn = 23.0% e) Mg = 0.2% f) Mg = 0.3% g) Zn = 1.8% h) Cr = 0.8% i) Cr = 0.8% j) Zn = 25.3%, Pb = 1.0% k) Zn = 9.8% l) Zn = 20.1%, Au = 0.3% m) Mg = 13.3% n) Mg = 8.8%, Cr = 0.6%

TableS 7-3 Results of S7-B sample long diameter (d), EDX analysis and materials

No, analy. spot	d (μm)	C (%)	O (%)	Al (%)	Si (%)	Cu (%)	Na (%)	Cl (%)	Others (%)	Materials
23, 1	36.4	8.6	0	0	0	59.1	0	0	32.3[a]	C
23, 2	36.4	26.4	0	0	0	46.9	0	0	26.7[b]	C
24, 1	2.5	67.6	23.3	9.1	0	0	0	0	0	E
25, 1	16.2	44.2	3.6	0	0	32.7	0	0	19.6[c]	C
26, 1	18.3	92.7	6.0	0	0	1.0	0	0	0.3[d]	C
27, 1	27.3	84.0	16.0	0	0	0	0	0	0	A
28, 1	3.0	27.0	58.9	11.8	0	0	0.7	0	1.6[e]	E
29, 1	6.0	38.5	50.3	9.4	0	0	0.4	0	1.4[f]*	E
30, 1	24.1	0	0	0	0	64.2	0	0	35.8[g]	C
31, 1	4.2	45.5	0	0	0	31.9	0	0	22.6[h]	C
32, 1	6.7	39.8	0	0	0	37.3	0	0	22.9[i]	C
33, 1	20.0	89.7	10.3	0	0	0	0	0	0	A
34, 1	1.5	92.6	5.7	0	0	1.2	0	0	0.5[j]	C
35, 1	13.8	94.7	3.7	0	0	1.6	0	0	0	C
36, 1	8.1	13.9	60.6	0	14.3	0	0	0	11.2[k]	B
36, 2	8.1	30.3	42.0	0	10.8	0	0	0	10.8[l]	B
37, 1	166.7	93.4	6.6	0	0	0	0	0	0	A
37, 2	166.7	92.8	7.2	0	0	0	0	0	0	A
37, 3	166.7	95.8	4.2	0	0	0	0	0	0	A

% = mol%. A: Non Si polymers. B: Si paints. C: Cu paints. D: Other paints. E: Al coating laminate films. F: Shell. *) MoS_2 lubricant contamination a) Zn = 32.3% b) Zn = 26.7% c) Zn = 19.1%, Pb= 0.5% d) Au = 0.3% e) S = 1.6% f) S = 1.1%, Mo = 0.4% g) Zn = 35.8% h) Zn = 22.6% i) Zn = 22.9% j) Zn = 0.5% k) Mg = 11.2% l) Mg = 11.2%

TableS 7-4 Results of S7-B sample long diameter (d), EDX analysis and materials

No, analy. spot	d (μm)	C (%)	O (%)	Al (%)	Si (%)	Cu (%)	Na (%)	Cl (%)	Others (%)	Materials
38, 1	5.6	72.3	3.0	0	0	15.7	0	0	9.1[a]	C
39, 1	5.2	51.1	3.9	0	0	27.8	0	0	17.2[b]	C
40, 1	9.3	77.9	20.8	0	0	0	0	0	1.3[c]	A

% = mol%. A: Non Si polymers. B: Si paints. C: Cu paints. D: Other paints. E: Al coating laminate films. F: Shell.
[a)] Zn = 9.1% [b)] Znr = 17.2% [c)] Znr = 1.3%

TableS 8-1 Results of S7-D sample long diameter (d), EDX analysis and materials

No, analy. spot	d (μm)	C (%)	O (%)	Al (%)	Si (%)	Cu (%)	Na (%)	Cl (%)	Others (%)	Materials
1, 1	26.3	33.8	39.3	26.	0	0	0	0	0	E
2, 1	8.0	68.4	26.7	0	2.7	0	0	0	2.2[a]	B
3, 1	3.4	44.3	41.9	0	7.7	0	0	0	6.1[b]	B
4, 1	7.3	66.3	24.0	0	5.7	0	0	0	4.0[c]	B
4, 2	7.3	29.6	51.7	0	10.3	0	0	0	8.4[d]	B
5, 1	6.0	48.3	43.8	6.5	0	0	0.3	0.3	0.8[e]	E
6, 1	3.8	53.1	40.5	5.7	0	0	0	0.2	0.7[f]	E
7, 1	4.5	47.5	43.7	7.4	0	0	0.5	0.4	0.6[g]	E
8, 1	6.5	50.0	42.2	6.6	0	0	0.4	0.3	0.5[h]	E
9, 1	4.0	38.0	51.6	8.6	0	0	0.5	0.3	1.0[i]	E
10, 1	7.5	65.6	3.2	0	0	19.2	0	0	12.1[j]	C
11, 1	3.7	55.8	2.8	0	0	26.3	0	0	15.0[k]	C
12, 1	1.7	72.8	3.2	0	0	14.9	0	0	9.1[l]	C
13, 1	7.0	38.7	2.1	0	0	38.0	0	0	21.3[m]	C
14, 1	2.0	81.1	7.9	0	0	6.6	0	0	4.4[n]	C
15, 1	10.0	75.7	9.1	0	1.7	6.9	0	0	6.6[o]	C
16, 1	10.0	74.8	25.2	0	0	0	0	0	0	A
17, 1	13.5	29.8	43.7	0	15.9	0	0	0	10.6[p]	B
17, 2	13.5	51.1	25.7	0	14.7	0	0	0	8.5[q]	B

% = mol%. A: Non Si polymers. B: Si paints. C: Cu paints. D: Other paints. E: Al coating laminate films. F: Shell.
a) Mg = 2.2% b) Mg = 7.1% c) Mg = 4.0% d) Mg = 8.4% e) S = 0.8% f) S = 0.7% g) S = 0.6% h) S = 0.5% i) S = 1.0% j) Zn = 12.1% k) Zn = 15.0% l) Zn = 8.4%, Pb = 0.7% m) Zn = 21.3% n) S = 0.3%, K = 0.5%, Zn = 3.6% o) K = 1.7%, Zn = 4.8% p) Mg = 10.6% q) Mg = 8.5%

TableS 8-2 Results of S7-D sample long diameter (d), EDX analysis and materials

No, analy. spot	d (μm)	C (%)	O (%)	Al (%)	Si (%)	Cu (%)	Na (%)	Cl (%)	Others (%)	Materials
18, 1	22.0	30.8	42.6	0	16.0	0	0	0	10.7[a]	B
19, 1	4.0	51.0	39.5	0	5.3	0	0	0	4.2[b]	B
20, 1	6.0	41.8	42.8	0	8.7	0	0	0	6.7[c]	B
21, 1	9.1	52.0	35.5	0	6.8	0	0	0	5.7[d]	B
21, 2	9.1	37.8	43.4	0	10.8	0	0	0	8.0[e]	B
22, 1	2.3	50.0	3.9	0	0	29.2	0	0	17.0[f]	C
23, 1	3.0	42.7	2.6	0	0	34.5	0	0	20.1[g]	C
24, 1	6.3	21.7	0	0	0	51.4	0	0	26.9[h]	C
24, 2	6.3	21.2	0	0	0	50.6	0	0	28.2[i]	C

% = mol%. A: Non Si polymers. B: Si paints. C: Cu paints. D: Other paints. E: Al coating laminate films. F: Shell. [*)] MoS_2 lubricant contamination [a)] Zn = 23.3% [b)] Mg = 4.2% [c)] Mg = 6.7% [d)] Mg = 5.3%, Cr = 0.4 [e)] Mg = 8.0% [f)] Zn = 16.1%, Pb =0.8% [g)] Zn = 19.5%, Au = 0.7% [h)] Zn = 26.9% [i)] Zn = 28.2%

TableS 8-3 Results of S7-D sample long diameter (d), EDX analysis and materials

No, analy. spot	d (μm)	C (%)	O (%)	Al (%)	Si (%)	Cu (%)	Na (%)	Cl (%)	Others (%)	Materials
25, 1	12.8	83.3	13.4	0	0	0	1.3	1.5	0.5[a]	A
25, 2	12.8	81.4	17.2	0	0	0	0.8	0.6	0	A
26, 1	17.5	39.3	34.2	12.5	13.1	0	0	0	1.1[b]	B
26, 2	17.5	38.1	35.8	11.8	12.8	0	0	0	1.4[c]	B
27, 1	20.0	94.1	4.0	0	1.5	0	0	0	0.3[d]	C
28, 1	16.0	94.0	5.8	0	0	0	0	0	0.3[e]	A
29, 1	15.0	80.3	3.0	0	10.8	0	0	0	6.0[f]	C
30, 1	23.3	46.9	2.8	0	31.8	0	0	0	18.6[g]	C
31, 1	5.7	72.0	5.4	0	0	14.5	0	0	8.1[h]	C
32, 1	3.6	63.3	2.8	0	0	21.2	0	0	12.7[i]	C
33, 1	7.0	47.5	2.1	0	0	32.0	0	0	18.4[j]	C
34, 1	10.0	63.6	3.0	0	0	20.8	0	0	12.6[k]	C
35, 1	6.4	30.9	5.3	0	0	39.0	0	0	24.7[l]	C
36, 1	17.9	92.3	7.7	0	0	0	0	0	0	A
37, 1	8.8	91.5	6.9	0	0	1.6	0	0	0	C
38, 1	5.0	58.6	17.0	2.8	4.7	6.4	0	0	10.6[m]	B
39, 1	18.3	94.1	5.9	0	0	0	0	0	0	A
40, 1	12.6	70.0	5.8	0	0	0	0	0.4	23.8[n]	D
40, 2	12.6	43.6	2.2		0	0	0	0	54.2[o]	D

% = mol%. A: Non Si polymers. B: Si paints. C: Cu paints. D: Other paints. E: Al coating laminate films. F: Shell.
a) Mg = 2.2% b) Mg = 0.4%, K = 0.6% c) K = 0.6%, Fe = 0.8% d) Au = 0.3% e) Au = 0.3% f) Zn = 6.0% g) Zn = 18.6% h) Zn = 8.1% i) Zn = 12.7% j) Zn = 18.4% k) Zn = 12.6% l) Zn = 21.9%, Pb = 2.9% m) Mg = 7.2%, Zn = 3.4% n) Ni = 23.8% o) Ni = 54.2%

TableS 8-4 Results of S7-D sample long diameter (d), EDX analysis and materials

No, analy. spot	d (μm)	C (%)	O (%)	Al (%)	Si (%)	Cu (%)	Na (%)	Cl (%)	Others (%)	Materials
41, 1	19.0	15.7	50.3	31.9	0	0	2.1	0	0	E
42, 1	18.3	62.3	29.6	1.0	2.7	0	1.4	1.1	2.1[a]	B
43, 1	10.0	85.4	14.0	0.3.	0.4	0	0	0	0	A

% = mol%. A: Non Si polymers. B: Si paints. C: Cu paints. D: Other paints. E: Al coating laminate films. F: Shell.

[a)] K = 0.8%, Fe = 1.3%

TableS 9-1 Results of S9-B sample long diameter (d), EDX analysis and materials

No, analy. spot	d (μm)	C (%)	O (%)	Al (%)	Si (%)	Cu (%)	Na (%)	Cl (%)	Others (%)	Materials
1, 1	8.7	36.7	0	0	0	63.3	0	0	0	C
2, 1	3.0	45.3	0	0	0	50.1	0	0	4.6[a]	C
3, 1	6.0	62.3	18.8	0	0	0	1.2	0	17.8[b]	D
4, 1	5.3	100	0	0	0	0	0	0	0	A
5, 1	0.7	65.2	4.5	0	0	18.1	0	0	12.3[c]	C
5, 2	0.7	51.3	0	0	0	30.8	0	0	17.9[d]	C
6, 1	2.3	41.7	9.7	0	0	28.3	0	0	20.3[e]	C
6, 2	2.3	35.0	0	0	0	41.1	0	0	23.9[f]	C
7, 1	5.4	77.1	0	0	0	15.9	0	0	7.0[g]	C
8, 2	6.2	46.8	2.4	0	0	32.0	0	0	18.8[h]	C
9, 3	7.6	62.9	0	0	0	23.0	0	0	14.1[i]	C
10, 1	5.2	0	0	0	0	64.1	0	0	36.0[j]	C
11, 1	19.5	0	7.1	0	0	51.6	0	0	41.4[k]	C
12, 1	38.0	78.8	14.3	2.1	0	0	0	4.8	0	E
13, 1	3.5	87.3	11.0	0	0.6	0	0	1.2	0	A
14, 1	7.4	67.4	6.2	0	16.3	0	0	0	11.1[l]	B
15, 1	34.3	44.7	0	0	12.2	0	0	0	8.9[m]	B
16, 1	5.8	13.0	1.3	0	0	54.5	0	0	31.2[n]	C
17, 1	8.5	23.2	3.1	0	0	47.2	0	0	26.6[o]	C
18, 1	9.0	29.0	4.0	0	0	39.1	0	0	27.9[p]	C

% = mol%. A: Non Si polymers. B: Si paints. C: Cu paints. D: Other paints. E: Al coating laminate films. F: Shell.
[a)] Mg = 4.6% [b)] S = 8.8%, Pb = 9.0% [c)] Zn = 12.3% [d)] Zn = 17.9% [e)] Zn = 19.6%, Pb = 0.6% [f)] Zn = 23.9% [g)] Zn = 7.0% [h)] Zn = 18.8% [i)] Zn = 14.1% [j)] Zn = 36.0% [k)] S=6.3, Zn = 27.5%, Pb = 7.6% [l)] Zn = 9.2%, Pb = 0.9% [m)] Mg = 8.9% [n)] Zn = 31.2% [o)] Zn = 26.6% [p)] Zn = 27.2%, Pb = 0.8%

TableS 9-2 Results of S9-B sample long diameter (d), EDX analysis and materials

No, analy. spot	d (μm)	C (%)	O (%)	Al (%)	Si (%)	Cu (%)	Na (%)	Cl (%)	Others (%)	Materials
19, 1	12.9	34.4	4.8	0	0	37.6	0	0	23.3[a]	C
20, 1	8.3	41.2	1.0	0	0	32.9	0	0	24.8[b]	C
21, 1	19.0	36.9	38.3	24.8	0	0	0	0	0	E
22, 1	17.5	10.5	36.1	0	0	0	0	0	53.4[c]	D*
23, 1	21.5	81.0	19.0	0	0	0	0	0	0	A
24, 1	9.1	0	0	0	0	62.2	0	0	37.8[d]	C
25, 1	3.1	16.4	0	0	0	53.0	0	0	30.6[e]	C
26, 1	4.3	25.5	0	0	0	45.9	0	0	28.6[f]	C
27, 1	2.0	68.6	27.0	0	2.6	0	0.5	0	1.4[g]	B
28, 1	8.0	83.7	14.9	0	0	0	0.8	0.6	0	A
29, 1	11.0	47.9	5.5	0	0	29.1	0	0	17.5[h]	C
29, 2	11.0	88.5	4.5	0	0	4.4	0	0	2.7[i]	C

% = mol%. A: Non Si polymers. B: Si paints. C: Cu paints. D: Other paints. E: Al coating laminate films. F: Shell.
*) MoS_2 lubricant contamination a) Zn = 23.3% b) S = 8.8%, Pb = 9.0% c) S = 12.4%, K = 8.8%, Fe = 26.2%, Mo = 5.8% d) Zn = 37.8% e) Zn = 30.6% f) Zn = 28.6% g) Zn = 7.0% h) Zn = 17.0%, Pb = 0.5% i) Zn = 2.7% = 0.8%

TableS 9-3 Results of S9-B sample long diameter (d), EDX analysis and materials

No, analy. spot	d (μm)	C (%)	O (%)	Al (%)	Si (%)	Cu (%)	Na (%)	Cl (%)	Others (%)	Materials
30, 1	4.3	57.6	34.8	0.3	6.8	0	0.6	0	0	B
31, 1	0.4	57.8	35.4	0.3	6.0	32.9	0.5	0	0	C
32, 1	6.6	19.3	55.2	0	0	0	3.5	0	21.9[a]	F*
33, 1	0.9	14.6	59.9	0	0	0	2.9	0	22.7[b]	F*
34, 1	1.8	44.2	6.4	0	0	29.7	0	0	19.7[c]	C
35, 1	1.3	15.3	2.0	0	0	47.8	0	0	34.8[d]	C
36, 1	38.4	78.2	19.6	0	0.4	0	0.5	0.4	1.0[e]	A
37, 1	48.5	59.4	3.1	0	0	0	17.4	20.	0	A
38, 1	30.8	69.9	11.5	0	0	10.1	5.4	1	0	C
39, 1	4.5	49.0	38.3	0.5	11.5	0	0.7	3.1	0	B

% = mol%. A: Non Si polymers. B: Si paints. C: Cu paints. D: Other paints. E: Al coating laminate films. F: Shell. *) MoS_2 lubricant contamination a) S = 8.0%, K = 1.2, Ca = 9.1, Mo = 3.6% b) S = 7.6%, K = 1.2, Ca = 9.8, Mo = 4.1% c) Zn = 19.7% d) Zn = 34.2%, Pb = 0.8% e) S = 0.7%, Ca = 0.3%

TableS 10-1 Results of S9-D sample long diameter (d), EDX analysis and materials

No, analy. spot	d (μm)	C (%)	O (%)	Al (%)	Si (%)	Cu (%)	Na (%)	Cl (%)	Others (%)	Materials
1, 1	8.8	11.9	0	0	0	54.2	0	0	33.8[a]	C
2, 1	3.1	11.1	64.5	0	0	50.1	0	0	24.3[b]	C*
3, 1	4.2	15.6	67.9	0	0	0	0	0	16.5[c]	D
4, 1	5.1	12.5	71.7	0	0	0	0	0	15.8[d]	D*
5, 1	4.5	29.1	4.1	0	0	41.4	0	0	25.4[e]	C
6, 1	5.3	0	2.7	0	0	63.2	0	0	34.1[f]	C
7, 1	5.0	50.1	3.7	0	0	27.8	0	0	18.5[g]	C
8, 1	4.5	59.4	4.2	0	0	23.3	0	0	13.1[h]	C
9, 1	3.4	30.1	3.0	0	0	42.2	0	0	24.7[i]	C
10, 1	3.1	47.9	3.3	0	0	30.7	0	0	18.2[j]	C
11, 1	1.0	78.1	18.1	0	0	0	1.5	1.2	1.0[k]	A
12, 1	24.0	78.5	18.3	0	0	0	0.9	0.7	1.7[l]	A
13, 1	4.0	60.2	30.5	0	0	2.7	0	0.5	6.2[m]	C
14, 1	10.0	12.1	57.2	0	17.6	0	0	0	13.1[n]	B
14, 2	10.0	23.3	54.3	0	12.5	0	0	0	10.0[o]	B
15, 1	21.2	77.2	17.2	0.5	1.7	0	1.1	0.9	1.4[p]	A
15, 2	21.2	80.1	15.8	0	0.8	0	0.4	1.1	1.8[q]	A

% = mol%. A: Non Si polymers. B: Si paints. C: Cu paints. D: Other paints. E: Al coating laminate films. F: Shell. [*)] MoS_2 lubricant contamination [a)] Zn = 33.8% [b)] S = 6.3%, K= 3.7%, Fe = 11.2%, Mo =3.2% [c)] S = 5.7%, K= 2.7%, Fe = 7.5%, Tl = 0.5% [d)] S = 4.6%, K= 2.5%, Fe = 7.0%, Mo =1.7% [e)] Zn = 23.3%, Au = 1.0, Pb = 1.1% [f)] Zn = 34.1% [g)] S = 0.4%, Zn = 17.5%, Pb = 0.5% [h)] Zn = 12.8%, Pb = 0.3% [i)] Zn = 24.7% [j)] Zn = 18.2% [k)] S=0.5, K = 0.5% [l)] S = 0.5%, K = 0.6%, Fe = 0.6 [m)] S = 1.6%, K = 0.8, Fe = 2.1, Zn = 1.7 [n)] Mg = 13.1% [o)] Mg = 10.0% [p)] S = 0.4%, K= 0.5%, Mg = 0.2%, P = 0.3% [q)] S = 0.6%, Zn= 1.2%, Tl = 0.1%

TableS 10-2 Results of S9-D sample long diameter (d), EDX analysis and materials

No, analy. spot	d (μm)	C (%)	O (%)	Al (%)	Si (%)	Cu (%)	Na (%)	Cl (%)	Others (%)	Materials
16, 1	2.5	70.4	4.5	0	0	15.8	0	0	9.3[a]	C
17, 1	7.0	63.2	3.6	0	0	20.9	0	0	12.3[b]	C
18, 1	5.0	29.1	2.1	0	0	44.2	0	0	24.6[c]	C
19, 1	10.0	9.4	0	0	0	53.4	0	0	37.1[d]	C
19, 2	10.0	14.8	0	0	0	49.1	0	0	36.1[e]	C
19, 3	10.0	13.0	0	0	0	55.0	0	0	32.0[f]	C
20, 1	100.0	18.3	55.4	0	0	0	0	0	26.3[g]	F
20, 2	100.0	19.4	57.8	0	0	0	0	0	22.8[h]	F
21, 1	68.6	19.5	61.7	0	0	0	0	0	18.8[i]	F
22.1	7.7	38.7	0	0	0	39.1	0	0	18.2[j]	C
22.2	7.7	63.8	6.9	0	0	18.1	0	0	11.1[k]	C
22.3	7.7	21.1	3.0	0	0	48.4	0	0	27.4[l]	C
22.4	7.7	70.8	16.5	0	0	5.0	0	0.4	7.4[m]	C
23, 1	7.0	63.1	1.6	0	22.8	0	0	0	12.6[n]	B
24, 1	5.7	13.3	0	0	56.2	0	0	0	30.5[o]	B
25, 1	4.1	24.5	4.0	0	46.1	0	0	0	25.4[p]	B

% = mol%. A: Non Si polymers. B: Si paints. C: Cu paints. D: Other paints. E: Al coating laminate films. F: Shell.
a) Zn = 9.3% b) Zn = 12.3% c) Zn = 24.6% d) Zn = 37.1% e) Zn = 36.1% f) Zn = 32.0% g) Ca = 26.3%
h) Ca = 22.8% i) Ca = 18.8% j) Zn = 22.1% k) Zn = 11.1% l) Zn = 27.4% m) S = 1.9%, K = 2.7%, Zn = 2.8%
n) Zn = 12.6% o) Zn = 30.5% p) Zn = 25.4%
